# Chemical Hazards in Products of Animal Origin in Cambodia from 2000 to 2023: A Systematic Review and Meta-Analysis

**DOI:** 10.3390/ijerph22081299

**Published:** 2025-08-19

**Authors:** Shwe Phue San, Linda Nicolaides, Delia Grace, Tumnoon Charaslertrangsi, Chhoun Chamnan, Shetty Seetharama Thombathu, Ra Thorng, Leab Kong, Sreymom Noeurn, Kuok Fidero, Che Ratana, Nazanin Zand, Rortana Chea

**Affiliations:** 1 Natural Resources Institute, University of Greenwich, Medway ME4 4TB, UK; ss6766m@greenwich.ac.uk (S.P.S.); l.nicolaides@greenwich.ac.uk (L.N.); n.zandfard@greenwich.ac.uk (N.Z.); 2 International Livestock Research Institute, Nairobi 00100, Kenya; 3 Science Division, Mahidol University International College, Nakhon Pathom 73170, Thailand; tumnoon.cha@mahidol.ac.th; 4 Fisheries Administration, Phnom Penh 120101, Cambodia; chhounchamnan@gmail.com (C.C.); sreymomnoeurn0908@gmail.com (S.N.); 5 United Nations Industrial Development Organization, Phnom Penh 120101, Cambodia; t.shetty@unido.org (S.S.T.); r.thorng@unido.org (R.T.); l.kong@unido.org (L.K.); 6 Ministry of Industry, Science, Technology, and Innovation, Phnom Penh 120203, Cambodia; kuok.fidero@misti.gov.kh; 7 Consumer Protection Competition and Freud Repression Directorate General, Phenom Penh 12125, Cambodia; cheratana@yahoo.com; 8 National Animal Health and Production Research Institute, General Directorate of Animal Health and Production, Phnom Penh 120603, Cambodia; rortanachea@gmail.com

**Keywords:** food contamination, chemical hazards, animal products, systematic review, meta-analysis, risk assessment, Cambodia

## Abstract

Chemical hazards in food present a significant health risk. The objective of our review is to understand health risks associated with chemical contaminants in products of animal origin (POAO) in Cambodia, where there is no known published study. We followed the “Preferred Reporting Items for Systematic Reviews and Meta-Analyses” (PRISMA) guidelines. A total of 23 reports were included for review. The findings are presented according to the PRISMA guidelines. The studies mostly focused on fishery products, with arsenic and mercury being the most frequently studied hazards. The evidence of banned substances such as chloramphenicol and certain organochlorine pesticides (OCPs), including chlordane and Mirex, was reported in fish and meat. Additionally, mercury levels were measured in beef, pork, viscera, and eggs, but the average concentration remained significantly below the hazard index. The average concentration of polycyclic aromatic hydrocarbons (PAH) in smoked fish exceeded the EU limits, ranging from 0.034 to 17.2 mg/kg, with an average mean concentration of 1.92 mg/kg. The pooled geometric means of arsenic and mercury in fish were 0.40 mg/kg (95% CI: 0.25–0.66) and ~0.14 mg/kg (95% CI: 0.087 to 0.223), respectively. The health risk of mercury contamination in fishery products needs the attention of the risk managers. However, industrial contaminants such as polybrominated diphenyl ethers (PBDEs) and butyl tin in marine fishes were lower than those reported elsewhere, such as Japan. We discuss the implications of the findings for human health and national food control systems (NFCS), the capacity of different agencies to undertake chemical risk assessment, the utility of systematic literature reviews (SLRs) for risk assessment and communication in low- and middle-income countries (LMICs), and the need for further research.

## 1. Introduction

Codex Alimentarius Commission (CAC) defines food hazard as a biological, chemical, or physical agent present in food that may lead to a negative health effect [1]. Chemical hazards in food include substances that can result in adverse health effects, regardless of their source, whether they come from natural origins or are introduced during food production and handling [2]. The presence of chemical contaminants in food is unintentional and undesirable, as these substances can pose health risks ranging from mild headaches to cancer when present at specific concentrations [3]. Foodborne chemicals consist of a wide range of substances, such as elemental contaminants, which include lead and cadmium, alongside persistent organic pollutants (POPs) such as organochlorine pesticides. Other examples of chemical contaminants in food include veterinary medical products (VMPs), food allergens, and non-food-grade food additives. Of the numerous chemical groups, naturally occurring toxins and environmental pollutants are particularly concerning for human health [4,5]. A variety of strategies are available to mitigate the risks associated with the chemical contaminants and to lower their concentrations in food, which include the enforcement of regulatory measures and the adoption of best practices. It is imperative to regulate the levels of chemical toxins in food products and minimize consumer exposure through food consumption. These regulatory frameworks, especially enforcing legislations and establishment of robust monitoring and surveillance systems, are critical elements of risk management approaches designed to tackle the issue of chemical contaminants that can occur at any stage of the food production chain, particularly in developing countries [4,6,7].

Preserving fish and meat poses significant food safety challenges in various parts of the world, including Cambodia, where there are limited facilities for cold chain management and the processing of perishable food items [8]. Polycyclic aromatic hydrocarbons (PAHs) are harmful compounds that are genotoxic, carcinogenic, and mutagenic, and they are generated when foods like fish or meat undergo smoking processes. Numerous studies have documented various concentrations of PAH4 (sum of benzo[a]pyrene (BaP), chrysene, benzo[b]fluoranthene, and benzo[a]anthracene) in smoked fish products globally. A study from Benin found the highest concentration of PAH4 at 0.48 ± 0.31 mg/kg in smoked dried fish. Heavy metals like cadmium, lead, and arsenic, which are toxic and carcinogenic, contaminate food through environmental pollution, with smoked fish often exceeding safety limits, particularly in African countries like Nigeria [9].

Heavy metals like mercury (Hg) and arsenic (As) pose significant food safety risks due to their toxicity, bioaccumulation, and persistence in the environment. Methylmercury (MeHg), a highly toxic organic form of mercury, accumulates in fish and seafood, leading to neurodevelopmental impairments in children and cardiovascular risks in adults. Inorganic arsenic, commonly found in groundwater, is linked to cancer, skin lesions, and cardiovascular diseases. Both metals enter food chains through industrial pollution, mining, and agricultural runoff, with climate change exacerbating their mobilization and uptake in crops and seafood [9]. A study conducted in China demonstrated that climate change amplifies methylmercury (MeHg) accumulation in freshwater wild fish, a critical yet underappreciated dietary MeHg source in Asia, projecting approximately a 60% rise in concentrations by mid-century under intermediate and high-emission scenarios. Regional disparities in exposure risks and associated economic losses highlight the need for integrated climate and mercury mitigation policies to address food safety inequalities and promote sustainable development [10].

The list of carcinogenic and non-carcinogenic chemicals commonly found in POAO is listed in the following Table 1.

There is an essential gap for Cambodia to implement a standardized monitoring system for chemical contamination throughout the entire food production chain to facilitate a comprehensive understanding of the specific contaminants responsible for public health issues and the pathways through which these contaminants infiltrate food systems. Such knowledge would enable the identification of key control points, thereby optimizing efforts to reduce contamination levels effectively [8]. In May 2001, Cambodia officially ratified the Stockholm Convention on Persistent Organic Pollutants (POPs), aiming to safeguard human health and the environment from the adverse effects of these substances. Subsequently, in 2006, the country formulated a National Implementation Plan (NIP) with support from the United Nations Environment Programme (UNEP), which was later revised in 2015 to correspond with UNEP’s updated inventory of POPs [19].

The challenges identified in previous studies in Cambodia included insufficient import control, inappropriate labeling practices, and the ongoing use of substances that are illegal or restricted [20]. While there are ongoing national initiatives, such as education programs to improve food safety and reduce chemical contamination in food production, particularly with respect to pesticides, Cambodia has still witnessed notable outbreaks related to pesticide and herbicide presence in food items [21]. Concerns regarding chemical toxins in food in Cambodia have been documented in earlier reports, particularly focusing on the use of fertilizers and pesticides aimed at boosting agricultural yields to accommodate the growing demand associated with population expansion and rising income levels. In addition, the Fisheries Administration (FiA) of Cambodia has initiated the implementation of the National Residue Monitoring Plan (NRMP) for farmed fish in ten provinces in Cambodia with the support of the European Union (EU) and United Nations Industrial Development Organization (UNIDO) since 2020. By contrast, there have been no known published studies on the compilation of different types of potential chemical toxins in POAO in Cambodia in the past two decades.

Our systematic review aims to evaluate the concentration levels of various chemical hazards, including environmental contaminants, persistent organic pollutants (POPs), and veterinary medical products (VMPs) in POAO in Cambodia over a 23-year period. In addition to analyzing contamination levels, the review compiles related data on study type, publication year, food source, and geographic location to identify knowledge gaps and priorities for strengthening Cambodia’s National Food Control System (NFCS). The specific objectives are (1) to identify reported chemical hazards in POAO; (2) to quantify concentrations of chemical toxins that exceed Cambodian, Codex Alimentarius Commission (CAC), and EU regulatory limits; and (3) to map contamination findings across different Cambodian provinces.

## 2. Materials and Methods

### 2.1. Protocol Development and Registration

The review protocol was developed according to the Preferred Reporting Items for Systematic Review and Meta-Analysis protocols (PRISMA-P) 2015 statement [22]. The review approach is identical to that applied for biological hazards in POAO in Cambodia [23]; however, the data concerning chemical hazards were analyzed separately. The concept of the protocol for chemical contaminants in POAO followed the previous review studies conducted by the International Livestock Research Institute (ILRI) for the Feed the Future Initiatives of the United States Agency for International Development (USAID) [24,25,26].

The PROSPERO registration number generated in March 2023 is PROSPERO 2023 CRD42023409476 and can be found at the weblink: https://www.crd.york.ac.uk/prospero/display_record.php?ID=CRD42023409476 (accessed on 27 March 2023).

The definitions of the key terms used in this review are listed in Abbreviations.

### 2.2. Eligibility Criteria

The review methodology followed the updated “PRISMA” guidelines available since 2020 [27].

#### 2.2.1. Inclusion Criteria

**Figure 1:**
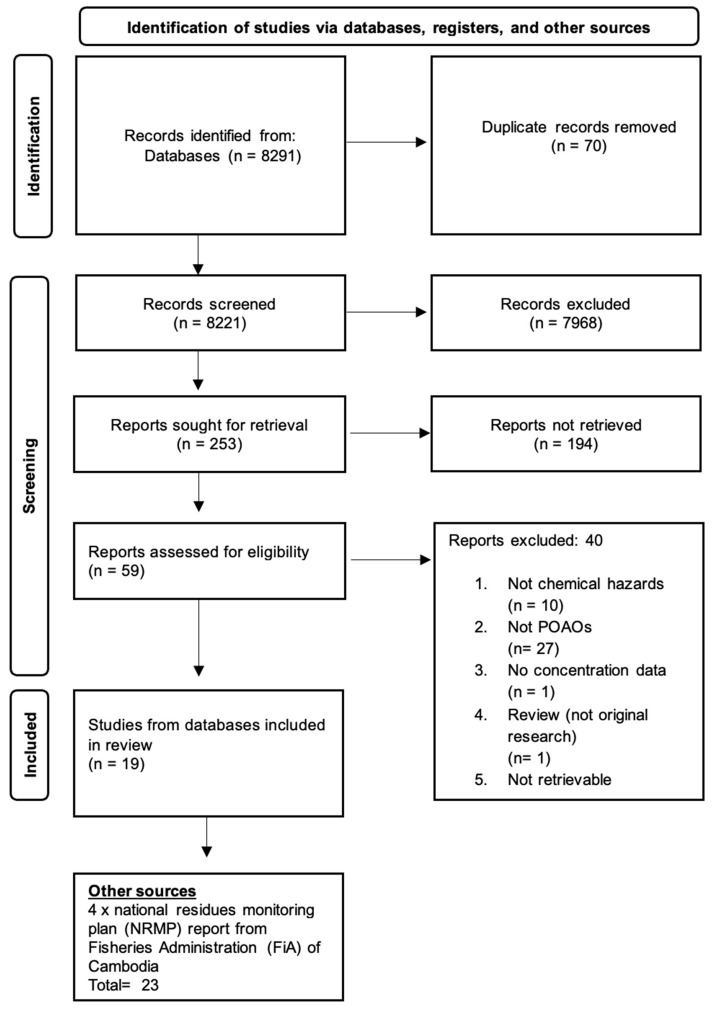
PRISMA flowchart showing identification, screening, and inclusion of eligible articles reporting chemical hazards in animals and POAO in Cambodia from 2000 to 2023.

The review established specific inclusion criteria, focusing on studies published in English within the timeframe of 1 January 2000 to 31 December 2022 from three academic databases, namely Scopus, PubMed, and Google Scholar. Additionally, reports from the NRMP sourced from the Fisheries Administration (FiA) between 1 January 2020 and 31 March 2024 were also included. The review included a variety of study types, such as exploratory studies, observational studies, and residue monitoring reports. Included studies were those that reported on the concentration of chemical hazards and provided related information, including sampling and testing methods, equipment employed, and the various stages of the production chain for food sourcing animals and different types of POAO. The reports retrieved from the chosen search databases and the FiA are presented in Figure 1.

#### 2.2.2. Exclusion Criteria

In conducting this review, certain exclusion criteria were applied. These included the omission of studies published in any language other than English, such as Khmer, as well as those that concentrated on non-foodborne hazards, biological hazards, and food types that were not classified as POAO. Additionally, laboratory studies that did not report on the concentration of chemical toxins, the equipment and methods utilized, or that involved populations outside of Cambodia were excluded. Studies that solely presented exposure data pertaining to humans were also not considered.

### 2.3. Databases and Search Strategy

Like the previous study [23], the search databases used for this review were Scopus, PubMed, and Google Scholar. The study selection included “(Foodborne OR “food borne” OR food-borne OR “food safety” OR “food related” OR “food associated” OR “food derived” OR “food * illness” OR “food * disease *” OR “food * intoxica *” OR “food pathogen” OR “food * poison *” OR “food * microb *” OR “food * vir *” OR “food parasit *” OR “food * toxin” OR “food * contamina *” OR “food * hazard *” AND (Cambodia *))”. Boolean operators (AND, OR, NOT or AND NOT) were used to combine or exclude the keywords in the search databases.

### 2.4. Screening and Study Selection

Results from three databases were compiled into a unified Excel sheet, and any duplicate studies were removed using Microsoft Excel. The initial step involved screening publication titles and abstracts in accordance with the inclusion and exclusion criteria specified in the review protocol. This screening was performed solely by the research student, with guidance from the supervisory team and external contributors. Following this, full papers associated with the accepted abstracts were sought and retrieved for the review.

Furthermore, the NRMP reports from the FiA that were submitted to the Directorate-General for Health and Food Safety of the European Commission (DG Sante) covering the period from the 2020 farming season to the 2023–2024 season were specifically requested for research purposes, and access to these documents was granted in August 2023.

### 2.5. Quality Assessment Criteria

To ensure the quality of each selected paper identified from the databases and all reports obtained from the FiA, the contents of the studies were assessed using the following four quality criteria questions.

- Is the method of study scientifically sound and clear?
- Is the laboratory testing method used for chemical contaminants in food appropriate?
- Are the descriptions of data analysis for key outputs (sampling, test methods, and concentration) accurate and precise?
- Are the outcomes and conclusions of the selected studies clearly written?

These quality assessment criteria were adapted from the previous systematic literature review (SLR) conducted by ILRI [23,24]. The studies were categorized into three classifications: “good,” “medium,” and “poor.” For data extraction, only those studies rated as good or medium were chosen (refer to Table 1). The selected studies were subsequently presented to the research team for evaluation and feedback.

### 2.6. Data Extraction

Following a thorough full-text screening, articles that were found to meet acceptable quality criteria were included for data extraction. The review concentrated on chemical contaminants present in food-sourced animals and POAO at any point in the production chain. The data extracted comprised of the type of food animal and POAO, the name and type of chemical contaminant, concentration details (including total sample counts, production chain type and stage, geographical context, sampling methods, analytical procedures, equipment used, and publication year), and the classification of the initiative (whether it was led by international institutions, involved joint efforts between national and international organizations, or was conducted by national institutions alone).

### 2.7. Data Analysis

#### 2.7.1. Meta-Analysis of Arsenic and Mercury Concentration in Fish [28,29,30]

For arsenic contamination in fish, we conducted a random-effects meta-analysis with the data extracted from the seven published papers [31,32,33,34,35,36,37]. The between-study variance (τ^2^) was estimated using Restricted Maximum Likelihood (REML), which is more robust than the traditional DerSimonian–Laird (DL) method, particularly when heterogeneity is high. To improve the accuracy of standard errors and confidence intervals, we applied the Knapp–Hartung adjustment, which is recommended in random-effects models with either few studies or high heterogeneity. Meta-regression was performed using random-effects models to explore potential sources of heterogeneity. Lastly, potential publication bias and small-study effects were assessed using funnel plots, trim and fit analysis, and the Egger test. Details of methods and findings can be accessed in Supplementary Materials File S2.

For mercury in fish, a pooled meta-analysis was conducted with the data extracted from 4 published papers [36,38,39,40] as the heterogeneity was estimable (I^2^, τ^2^), and random-effects model was used to reflect between-study variability. Meta-regression was not conducted as it needs at least 10 studies per predictor to avoid overfitting and spurious associations. Details methods and findings of the meta-analysis for mercury in fish can be accessed in Supplementary Materials File S3.

#### 2.7.2. Descriptive and Basic Statistical Analysis

Other than arsenic and mercury present in fish, the remaining chemical contaminants could not undergo meta-analysis due to limited data availability, including missing values of standard deviations and ranges, and were therefore primarily analyzed descriptively. However, the basic statistics were calculated for each of the remaining chemical contaminants in Microsoft Excel for the values of minimum, maximum, average, and percentage of total number of samples exceeded the maximum residue limits set by the CAC and the EU. Hazards indexes (HIs) for lead and mercury detected in meat (beef, beef viscera, and pork), eggs, and fish were calculated according to the data availability and estimates as stated in Section 2.8.

### 2.8. Health Risk Assessment of Chemical Toxins in Fishery Products

Health risk assessments for cadmium and mercury were performed following methodologies applied in previous studies [41,42], as adequate data for these contaminants were obtained from the studies included in our review. The findings are presented in Section 3.5.3. To evaluate the toxicological implications of human exposure to the chemical contaminants identified in POAO, it is crucial to calculate the estimated weekly intake (EWI) of environmental contaminants and compare it with the tolerable weekly intake (TWI) set by the European Food Safety Authority (EFSA). The human health risk assessments were based on the TWI database and the estimated annual per capita consumption of POAO, assuming an average body weight of 60 kg for the Cambodian population. The estimated annual fish consumption in Cambodia is 63 kg per capita, which includes 44.2 kg of inland fish and 17.3 kg of marine fish [43]. According to the report of the General Directorate of Animal Health and Production (GDAHP), the annual consumption of meat per capita per year in 2024 was 18.5 kg, consisting of 5.15 kg of beef and water buffalo, 9.35 kg of pork, 3.95 kg of poultry, and 0.05 kg of lamb and goat meat.

The European Food Safety Authority established the tolerable weekly intake (TWI) of cadmium and mercury as 2.5 and 4 μg/kg body weight, respectively. The results obtained were utilized to calculate the EWI, expressed in μg/kg body weight/day, using the following equation:

EWI = C × F/W (1)

where:

C = concentration of the contaminant (μg/kg);
F = weekly food consumption per person (kg);
W = mean body weight (60 kg).

The evaluation of health risk was conducted using the hazard index (HI), which was determined by calculating the ratio of estimated weekly intake (EWI) to the tolerable weekly intake (TWI) values as follows.

HI = Estimated weekly intake (μg/kg bw)/Tolerable weekly intake (μg/kg bw)(2)

### 2.9. Calculation of DALY/Population of Cambodia

The calculation of foodborne illnesses, deaths, and disability-adjusted life years (DALY) caused by certain chemical toxins and contaminants per population in Cambodia was based on the estimation provided by the WHO [44,45]. The population of Cambodia, according to the World Bank data estimated in 2022, was 16,767,842 [46]. The DALYs for the Cambodian population for metals, namely arsenic, cadmium, lead, and mercury, as well as other toxins such as aflatoxin, cyanide in cassava, and dioxin were stated further on.

## 3. Results

As shown in Figure 1, a total of 8291 records were retrieved from three academic databases and the residue monitoring reports of FiA, as stated earlier. After the removal of duplicates using Excel, the number of records available for screening decreased to 8221. During the title screening phase, 7968 records were eliminated due to their irrelevance to food safety hazards. This process led to the assessment of 253 abstracts, resulting in the exclusion of 194 reports that pertained to human cases rather than food-sourced animals or POAO. Ultimately, 19 records underwent a comprehensive review, while 40 records were discarded for various reasons detailed in Figure 1. The list of records included in the review is presented in Supplementary Materials File S1. Additionally, four reports were obtained with the consent of the FiA and were reviewed for data extraction.

The evidence of chemical hazards in products of animal origin in Cambodia from 2000 to 2024, reported by 23 studies included in the review, was summarized in Table 2. Likewise, we summarized the evidence of concentration of chemical contaminants in different POAO in Cambodia from 2000 to 2024 in terms of minimum, maximum, average, and percentage of non-compliances against the maximum residue levels established by the CAC and the EU.

### 3.1. Type of Study Initiatives

**Figure 2:**
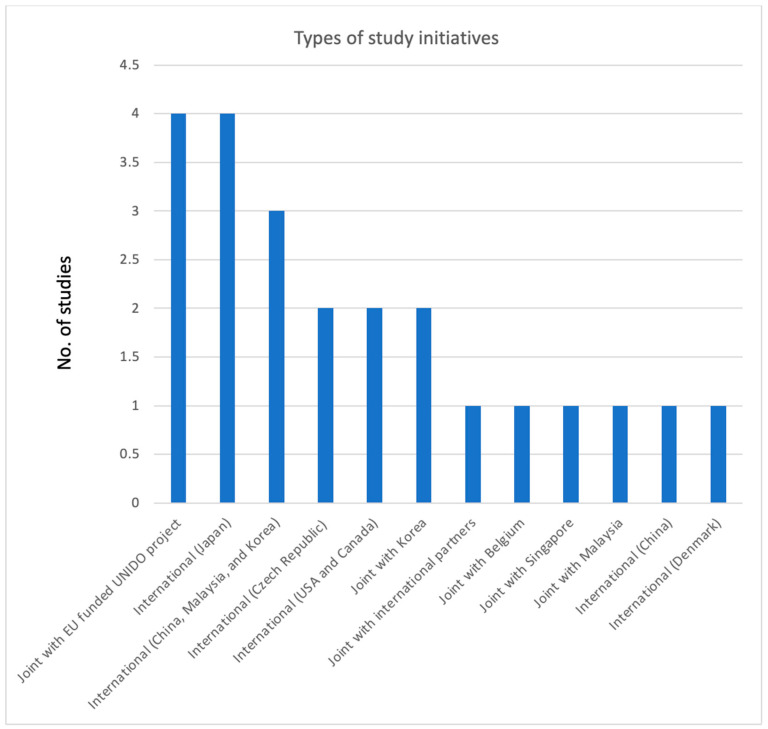
Evidence of study frequencies for chemical contamination in animals and POAO in Cambodia conducted by a joint initiative, and international institutions from 2000 to 2023.

Figure 2 illustrates that there is a lack of evidence indicating that national research institutions conducted studies independently, without the collaboration of international partners for funding or technical assistance. Conversely, the establishment of bilateral or multilateral partnerships between national and international entities resulted in the execution of ten studies, which benefited from the financial or technical support of these collaborators. Among the bilateral studies, those associated with the EU-funded CAPFISH-Capture: Postharvest Fisheries Development project implemented by UNIDO had the highest frequencies, followed by collaborations with the Republic of Korea. The UNIDO initiative facilitated the FiA in executing the NRMP and in preparing the report for submission to the DG Sante of the European Commission for the EU market access for farmed fish from Cambodia. The NRMP studies focused on the presence of veterinary drug residues, pesticides, and contaminants, including persistent organic pollutants and metals, in freshwater farmed fish intended for the EU market. Furthermore, the joint studies included contributions from Belgium, Singapore, and Malaysia, as well as multiple international partners. In contrast, four studies were conducted by research institutions in Japan, three were jointly executed by China, Korea, and Malaysia, two studies were reported collaboratively by the USA and Canada, two studies were reported by the research institutions in the Czech Republic, and one study each was conducted by China and Denmark. In contrast, our review found no indications of bilateral research partnerships between Cambodia and its neighboring countries, namely Thailand, the Lao PDR, and Vietnam.

### 3.2. Number of Reviewed Studies and Geographical Coverage

**Figure 3:**
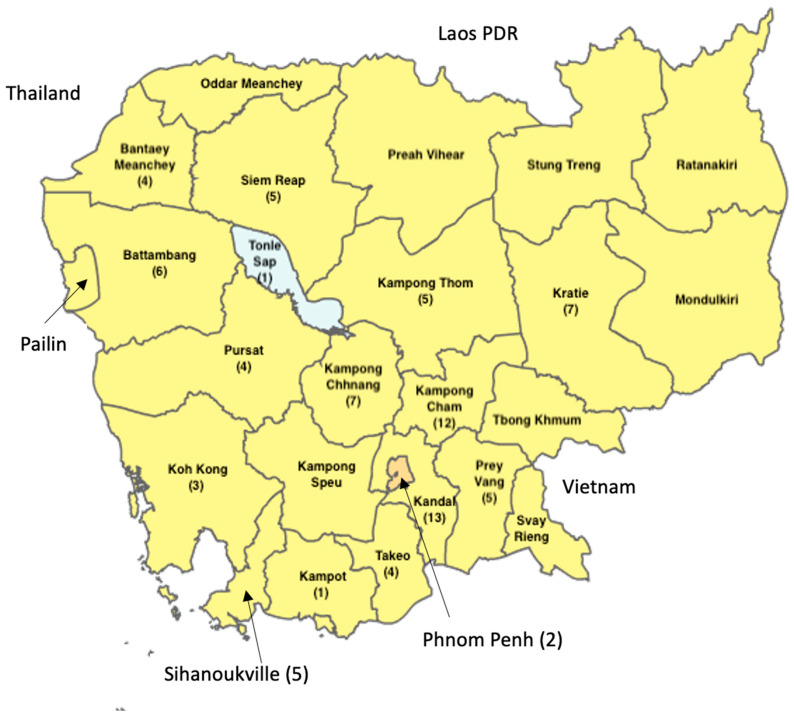
Frequency of studies on chemical contaminants in certain provinces in Cambodia from 2000 to 2023.

As shown in Figure 3, Kandal province had the largest number of studies included in the review, with Kampong Chhnang following closely behind. In contrast to the biological hazards found in POAO [23] in Cambodia, only two studies were conducted in Phnom Penh, the capital of Cambodia; however, Kandal is the nearest province to Phnom Penh. Over the past two decades in Cambodia, we did not identify any evidence of published research concerning chemical contaminants in food across nine out of the 25 provinces. These provinces included Pailin, Oddar Meanchey, Preah Vihear, Stung Trung, Ratanakiri, Mondulkiri, Tbong Khmum, Svay Rieng, and Kep. The evidence regarding chemical contamination in POAO studies was not comprehensive, as it did not cover all provinces of the entire country, particularly the plateau region and the border provinces adjacent to the Lao PDR, Thailand, and Vietnam.

### 3.3. Number of Publications and Reports from 2000 to 2023

**Figure 4:**
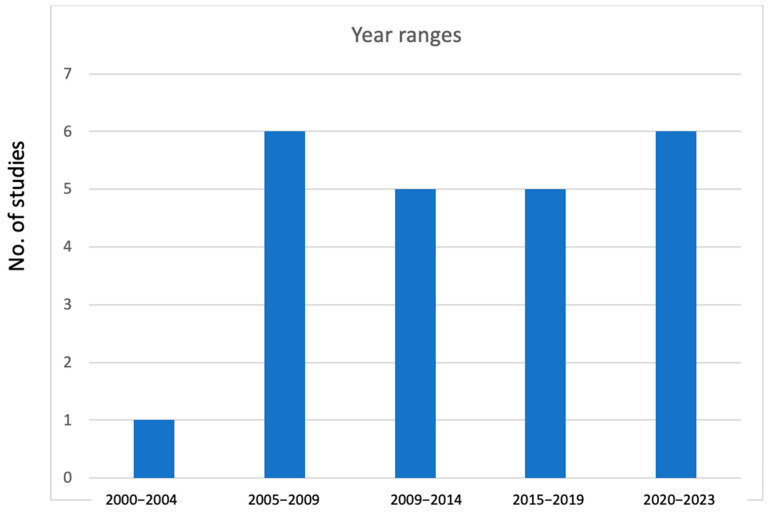
Number of publications of chemical hazards in POAO in Cambodia for different intervals from 2000 to 2023.

Figure 4 illustrates that only one study conducted in 2002 focused on monitoring butyl tin contamination in coastal waters was identified from the 2000 to 2004 period. The period from 2005 to 2009 marked a peak in the frequency of such studies, followed by a slight decrease during the intervals of 2010–2014 and 2015–2019. Over the subsequent four years, there was an increase in the number of studies addressing chemical contamination, with expectations of further growth by the end of 2024. The year 2020 marked the beginning of NRMP studies aimed at investigating veterinary drug residues, pesticides, and environmental contaminants, such as persistent organic pollutants (POPs) and metals, in farmed fish, driven by a partnership among public institutions, including the FiA.

### 3.4. Number of Studies for Different Groups of Chemical Contaminants

**Figure 5:**
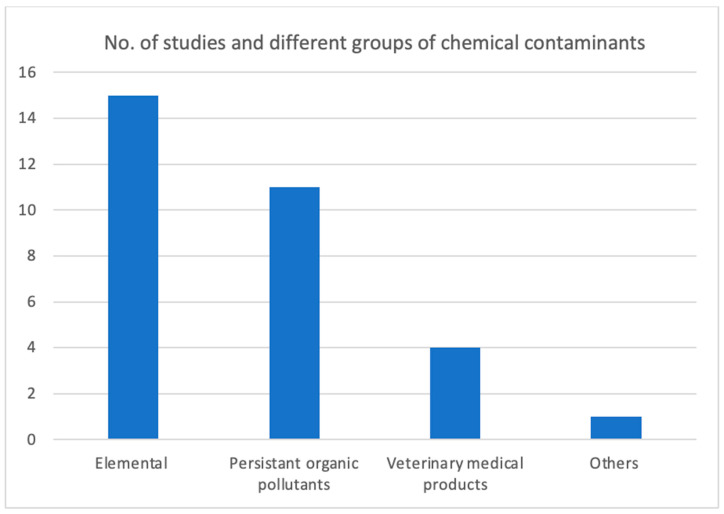
Evidence of frequency of studies identified for different groups of chemical hazards in POAO in Cambodia from 2000 to 2023.

The data presented in Figure 5 indicates that 15 of the 23 studies and reports addressed elemental toxins, specifically arsenic, cadmium, lead, and mercury. Meanwhile, 11 studies and reports focused on various toxins identified as persistent organic pollutants (POPs), which cover organochlorine pesticides, polycyclic aromatic hydrocarbons (PAHs), and butyl tin. Moreover, the review also included findings on the occurrence of residues of various veterinary medical products in farmed fish across 10 provinces in Cambodia. Nonetheless, the scope of veterinary medical products within POAO was restricted to farmed fish, namely tilapia, catfish, and snakehead, in ten provinces of Cambodia, while evidence of other essential meat products such as pork, chicken, and beef was not observed in the review. A cross-sectional study conducted in 2016, which examined dietary exposure and human risk assessment of phthalate esters in fish, meat, and viscera in Cambodia, was also incorporated into the review. We did not identify studies of other potential contaminants in POAO, such as mycotoxins, dioxins, chlorates, and so on.

### 3.5. Summary of Evidence of Chemical Contaminants in POAO

#### 3.5.1. Findings from Meta-Analysis of Arsenic (Meta-Regression)

The pooled geometric mean arsenic concentration in fish was 0.40 mg/kg (95% CI: 0.25–0.66). The 95% prediction interval ranged from 0.015 to 10.50 mg/kg, indicating that a future study under similar conditions could plausibly report arsenic concentrations anywhere in this range, consistent with extensive between-study heterogeneity. The results of heterogeneity statistics supported and confirmed extreme between-study variability, beyond what would be expected by chance alone.

#### 3.5.2. Findings from Meta-Analysis of Mercury (Pooled Concentration)

The average mercury level in fish is ~0.14 mg/kg (95% CI: 0.087 to 0.223), significantly above zero, and it serves as a meaningful finding for risk or regulation. Large τ^2^ suggesting large between-study variance (log scale) and high I2 95% of the total variability is between studies. Cochran’s Q confirms significant heterogeneity. There is substantial unexplained variability across studies, suggesting differences in species, sites, methods, or other study-level characteristics. In a future study, the mercury level could plausibly fall anywhere between 0.012 and 1.65 mg/kg—a very wide range, consistent with the high heterogeneity. Most studies carry ~3% weight, indicating a balanced sample. No single study dominates the result, but the high τ^2^ reduces precision.

#### 3.5.3. Descriptive and Basic Statistical Analysis of the Results

A summary of various chemical contaminants found in POAO was presented, detailing the names of hazards, food sources, sampling locations, and specific sampling points. Additionally, the total number of samples, testing methodologies, and equipment utilized were outlined in Table 3. The concentrations of each identified chemical hazard in the food were further analyzed, providing minimum, maximum, and average values. Furthermore, the percentage of non-compliance with the maximum residue limits established by the CAC and the EU was also included in Table 4.

### 3.6. Evidence of Elemental Contaminants Detected in POAO

In total, 15 out of 23 studies included in our review focused on different types of elemental toxins, specifically arsenic, cadmium, lead, and mercury, in various POAO, including beef, beef viscera, egg, fish, and pork. Under the group of elements, 1283 samples were tested, and details of each contaminant are as described below.

#### 3.6.1. Arsenic

The evidence of concentrations of arsenic and related information was reported in seven out of 19 published papers (Papers 1, 2, 10, 14, 15, 16, and 18 of Supplementary Materials S1) [31,32,33,34,35,37,47]. The 331 samples were taken from snails, clams, fish, beef, beef viscera, and pork. The sampling locations were aquaculture sites, Tonle Sap Lake, ponds, and rivers, as well as markets in Kampong Cham, Kratie, and Kandal provinces. For the laboratory analysis, acid digestion methods and internal procedures were applied by using different equipment with reliable accuracy and sensitivity, such as electrothermal atomic absorption spectrometry (ETAAS), Inductively Coupled Plasma Mass Spectrometry (ICP-MS), Inductively Coupled Plasma Optical Emission Spectrometry (ICP-OES), and high-performance liquid chromatography (HPLC)-ICP-MS.

In total, seven studies (Papers 1, 2, 10, 14, 15, 16, and 18) documented varying concentrations of arsenic in fish. Paper 10 provided data on arsenic concentration based on dry weight, while Paper 15 presented findings based on wet weight. The remaining studies did not provide specific details. Paper 14 indicated that the sample tissues were collected from both skin and flesh. Additionally, Paper 18 stated that analyses were conducted on muscle, liver, and skin tissues. According to the results of meta-analysis, the pooled geometric mean arsenic concentration in fish was 0.40 mg/kg (95% CI: 0.25–0.66). Details of meta regression analysis can be found in Supplementary Materials S2. Resulting from the basic statistical analysis, the concentrations of arsenic in beef, beef viscera, and egg were shown in Table 4. Despite the maximum limits of arsenic in other products such as rice and salt, the Codex Alimentarius Commission (CAC) and the EU do not have a specific maximum limit of arsenic in fish.

#### 3.6.2. Cadmium

In our review, Papers 9, 10, 14, and 18 [35,36,37,47] identified cadmium contamination in POAO. A total of 208 samples were gathered from both freshwater and marine environments in coastal regions, including Phnom Penh and the Tonle Sap Lake area. The acid digestion method was employed to assess cadmium levels in food, utilizing ETAAS and ICP-MS techniques. The concentration of cadmium in fishery products varied from 0.0003 to 0.035 mg/kg, with an average concentration of 0.28 mg/kg. The CAC and EU have established the maximum level of cadmium in fish as 0.05 mg/kg. Our review indicates that 9.09% of the samples tested did not meet the standards set by the CAC and the EU.

#### 3.6.3. Lead

The concentration and related information of lead in POAO were reported in Papers 9, 10, 14, and 18 of published databases and NRMP reports of the FiA [35,36,37,47]. A total of 249 freshwater fish samples were collected from various locations, including Phnom Penh, Tonle Sap Lake, Kandal, Prey Veng, Takeo, Kampong Cham, Kampong Chhnang, Pursat, Battambang, Kampong Thom, Siem Reap, and Banteay Meanchey. The laboratory analysis employed the acid digestion technique in conjunction with ICP-MS equipment. The lead concentration in the fish samples varied from below the limit of detection (LOD) to 0.31 mg/kg, with an average concentration of 0.059 mg/kg. The maximum limit of lead in fish established by the CAC and EU is 0.3 mg/kg. Notably, 5.88% of the samples failed to meet the standards set by the CAC and the EU.

#### 3.6.4. Mercury

The mercury contamination in POAO in Cambodia was reported in Papers 7, 9, 10, 12, 19, [36,38,39,40,47] and NRMP reports of the FiA. A total of 495 samples were gathered from various locations, including coastal waters, fish farms, and markets in Phnom Penh, Tonle Sap Lake, Kandal, Prey Veng, Takeo, Kampong Cham, Kampong Chhnang, Pursat, Battambang, Kampong Thom, Siem Reap, and Banteay Meanchey. For laboratory analysis, the acid digestion method was employed alongside techniques outlined by the United States Environmental Protection Agency (USEPA) under method no. 7473, utilizing AAS, direct mercury analysers, and ICP-MS equipment.

The four studies (Papers 7, 10, 12, and 19) reported the concentration of mercury in fish. Paper 10 documented the mercury concentration based on dry weight, while papers seven and 19 provided their findings in terms of wet weight. The results of meta-analysis revealed the pooled estimate of average mercury in fish is ~0.14 mg/kg (95% CI: 0.087 to 0.223). The concentrations of mercury in mg/kg for beef, beef viscera, eggs, and pork are summarized in Table 4. In total, 1.75% of the samples tested for mercury did not meet the CAC and EU standards.

### 3.7. Evidence of POPs Reported in POAO in Cambodia from 2000 to 2023

In our review, seven studies [48,49,50,51,52,53,54] reported the different types of POPs, consisting of PAH, polybrominated diphenyl ethers (PBDEs), butyl tin, and organochlorine pesticides. In total, 1130 samples were tested under the group of POPs, particularly for Benzo[*a*]pyrene (BaP), summation of 4 and 12 PAH (ƩPAH4 and ƩPAH12), summation of organochlorine pesticides (OCPs), hexachlorobenzene (HCB), hexachlorocyclohexane isomers (HCHs), PBDEs, total butyl tin and tri butyl tin. The detailed findings are elaborated below.

#### 3.7.1. Polycyclic Aromatic Hydrocarbons (PAH)

In our review, four papers reported the concentration of PAH in POAO specifically in beef, beef viscera, clams, eggs, fish, pork, and snails [50,51,52,53]. A total of 297 samples were obtained from processing sites and various locations.

##### Benzo[*a*]pyrene (BaP)

Benzo[*a*]pyrene (BaP) is a PAH, and three studies separately reported the concentrations of BaP in processed fishery products [50,52,53]. A total of 105 samples were obtained from fishery processing sites and markets in the provinces of Battambang, Kampong Cham, Kampong Chhnang, Kandal, and Siem Reap. The analysis of these samples utilized accelerated solvent extraction alongside modified Quick, Easy, Cheap, Effective, Rugged, and Safe (QuEChERS) methods, utilizing Gas Chromatography–Mass Spectrometry (GC/MS) and High-Performance Liquid Chromatography with a fluorescence detector (HPLC-FLD). The concentration levels of BaP were found to range from 0.005 to 0.9 mg/kg, with an average concentration of 0.13 mg/kg. It is found that 94.29% of the samples did not comply with the standards established by the CAC, which allows a maximum concentration of 0.005 mg/kg, while all samples (100%) surpassed the EU’s permissible limit of 0.002 mg/kg.

##### Sum of Polycyclic Aromatic Hydrocarbon 4 (ƩPAH4)

The sum of 4 PAH (ƩPAH4) included the combination of BaP, Chrysene, Benzo[α]anthracene, Benzo[β]fluoranthene, and three studies separately reported the concentrations of BaP in processed fishery products [50,52,53]. A total of 105 samples were collected from fishery processing sites and markets across the provinces of Battambang, Kampong Cham, Kampong Chhnang, Kandal, and Siem Reap. The analysis employed accelerated solvent extraction in conjunction with modified QuEChERS techniques, utilizing Gas Chromatography–Mass Spectrometry (GC/MS) and High-Performance Liquid Chromatography with a fluorescence detector (HPLC-FLD). The BaP concentration levels varied from 0.034 to 17.2 mg/kg, with an average concentration of 1.92 mg/kg. While the CAC does not establish a specific maximum limit for ƩPAH4, all samples analyzed failed to meet the EU’s threshold of 0.012 mg/kg.

##### Sum of Polycyclic Aromatic Hydrocarbon 12 (ƩPAH12)

The sum of 12 different PAH (ƩPAH12) consists of the sum of Fluorene, Phenanthrene, Anthracene, Fluoranthene, Pyrene, Chrysene, Benzo[a]anthracene, Benzo[b]fluoranthene, Benzo[a]pyrene, Indeno[1,2,3-cd]pyrene, Dibenzo[a,h]anthracene, and Benzo[ghi]perylene. In our review, two studies reported the concentration of ƩPAH12 [51,52]. A total of over 87 samples comprising mussels and smoked freshwater fish were gathered from various locations, including wharf walls, buoys, rocks, and processing sites in Battambang, Kampong Cham, Kampong Chhnang, Kandal, and Koh Kong. Laboratory analyses were conducted utilizing modified QuEChERS methodologies in conjunction with GC-MS instrumentation. The concentration of ƩPAH12 varied between 0.021 and 17.2 mg/kg, with an average concentration of 4.05 mg/kg. Neither the CAC nor the EU has any specific limits for the concentration of ƩPAH12 in fish and fishery products.

#### 3.7.2. Other Persistent Organic Pollutants (POPs)

In addition to PAH, our review identified other chemical contaminants under the category of POPs. These include butyl tin, organochlorine pesticides, and polybrominated diphenyl ethers (PBDEs) as follows.

##### Evidence of Butyl Tin in Fishery Products

A study included in the review reported the concentration of two types of butyl tin, namely total butyl tin and tributyl tin, in marine fish [49]. This study was conducted in Cambodia as part of the Asia-Pacific mussel watch program to monitor the contamination of butyl tin in coastal waters of developing countries in Asia, but not specifically for the purposes of food contamination. A total of 419 marine fish specimens were collected from Koh Kong and Sihanoukville, which included fishery sites, international harbor, and commercial harbors. For the analysis of these samples, a modified lipid extraction method was employed, utilizing GC-FPD as the analytical equipment. The concentration of total butyl tin (expressed in wet weight) varied between 0.0024 and 0.15 mg/kg, with an average concentration of 0.027 mg/kg. Similarly, the levels of tri-butyl tin in the marine fish samples ranged from 0.0024 to 0.088 mg/kg, yielding an average concentration of 0.015 mg/kg. It is noteworthy that neither the CAC nor the EU has established a maximum permissible limit for butyl tin in fish.

##### Evidence of POPs (PBDE and Organochlorine Pesticides) in POAO

In our review, a study reported the concentration of PBDE in 46 mussels samples collected from the coastal areas, namely Koh Kong and Sihanoukville. The lipid extraction method was utilized by using the electron capture detector in the GC (GC-ECD) [48]. Two studies revealed the concentration of organochlorine pesticides in 138 fish and mussel samples collected from Koh Kong, Sihanoukville, Kampong Cham, Kratie, and Kandal provinces (Table 5). For testing the samples, USEPA methods 3620B and 3640 A were employed by using GC-MS equipment [48,54].

### 3.8. Evidence of Residues of Veterinary Medicines in Farmed Fish

We conducted a review of the NRMP reports [55,56,57,58] of the FiA submitted to the DG-Sante of the EU annually since 2020 until the 2023 fish farming seasons. These reports adhered to the standard template established by the EU for the purposes of sampling, testing, and reporting. From 2020 to 2023, muscle tissues of 129 freshwater farmed fish samples were analyzed at internationally recognized food testing ^1^laboratories by using LC-MS-MS equipment for the presence of banned substances such as Chloramphenicol in three different varieties of freshwater farmed fish namely Pangasius catfish, snakehead, and tilapia from the fish farms in Kandal, Prey Veng, Takeo, Kampong Cham, Kampong Chhnang, Pursat, Battambang, Kampong Thom, Siem Reap, and Banteay Meanchey provinces. The concentration of chloramphenicol in farmed fish ranged from 0.03 to 1.18 mg/kg, with an average concentration of 0.255 mg/kg. In total, 23 out of 129 samples tested did not comply with national legislation and the requirements of the CAC and the EU. During the 2022 fish farming season, three pangasius catfish samples were found to be contaminated with other banned substances like nitrofuran metabolites (0.19 mg/kg in Kampong Chhnang and 0.14 mg/kg in Takeo province) and leuco-malachite green 1.46 mg/kg in Kandal province.

### 3.9. Evidence of Other Chemical Contaminants in POAO

Our review identified a study that reported the concentration of phthalate esters [59] in POAO, specifically beef, beef viscera, fish, and pork. A total of 42 samples were gathered from the farms and markets in Kampong Cham, Kratie, and Kandal provinces. The GC-MS equipment was used by employing the USEPA 3540C and 3620B methods. The concentration of phthalate esters in beef, beef viscera, fish, and pork is summarized in Table 4. Neither the CAC nor the EU achieved the maximum limits of phthalate esters in POAO.

### 3.10. Health Risk Assessment of Exposure to Cadmium and Mercury in POAO

As shown in Table 6, none of the hazard index of the average chemical toxin concentration in POAO exceeded 1, which could lead to a potential health threat. However, mercury concentrations in fish were the highest among other types of POAO, such as beef, beef viscera, egg, and pork. The mercury levels reached 1. These findings suggest a potential health risk associated with fish consumption and highlight the need for more comprehensive research on this issue.

## 4. Discussion

The evidence of our review indicated that Cambodia has effectively engaged with international research institutions to investigate chemical toxins in POAO over the past 23 years. However, this collaboration has predominantly with the international partners, with limited involvement from regional stakeholders in the Southeast Asia region (Figure 2). Aside from Malaysia and Singapore, there has been an absence of research collaboration with other immediate neighboring countries, Thailand, Vietnam, and the Lao PDR. The results suggest that food control authorities should embark on their own research initiatives concerning chemical contaminants in POAO. As shown in Figure 3, most studies have been concentrated in provinces surrounding Phnom Penh, the capital of Cambodia, likely due to the area’s high population density and economic activities, which aligns with previous research findings in Burkina Faso and studies on biological hazards in Cambodia [23,24].

The scope of our review covered the entire country; however, we found that research on chemical toxins in POAO has not been undertaken in 9 of the 25 provinces in Cambodia, revealing a considerable gap in the existing literature. These provinces are in the plateau region and are adjacent to neighboring countries. Given the substantial volume of informal trade between Cambodia and its neighbors, particularly Thailand and Vietnam [60], it is surprising that studies focusing on risk-based food import control measures have not been established yet. In contrast to the increasing research on biological hazards in Cambodia, investigations into food chemical toxins in POAO have not seen a significant rise recently over the period of two decades (Figure 4), likely due to the limited national analytical infrastructure and a lack of interest from international or regional collaborators.

Our findings (Figure 5) indicated that not all studies included in our review concentrated on food safety-related issues; specifically, three out of the nineteen published studies were directed towards environmental monitoring and assessment initiatives [48,49,51]. Nonetheless, these environmental monitoring programs provided indirect advantages to the country’s food control system, highlighting the necessity and significance of implementing the “One Health Approach.” In our review, we categorized chemical toxins in food into three distinct groups: elemental toxins, which include arsenic, cadmium, lead, and mercury; persistent organic pollutants (POPs), which encompass butyl tin, organochlorine pesticides, polycyclic aromatic hydrocarbons (PAHs), and polybrominated diphenyl ethers (PBDEs); and veterinary medicinal products (VMPs), which align with the EU’s NRMP requirements. It is noteworthy that there is a lack of known or published research concerning processing-related toxins in POAO, such as chlorates found in fish and other seafood, which arise from the improper application of chlorine-based sanitizing agents during processing.

Toxic elements represent a significant health risk among various contaminants due to their nature as non-biodegradable xenobiotics, which can persist in the environment and ultimately enter the food chain. The consumption of contaminated food is a primary pathway for exposure to these hazardous metals [42]. Given the significant fish consumption in Cambodia, there is a pressing need for more comprehensive studies on arsenic contamination in fish to facilitate both quantitative and qualitative risk assessments. Our review indicates that the highest arsenic concentration found in freshwater fish was 10.7 mg/kg, which is notably lower than the levels detected in snakehead fish, which ranged from 13.1 to 22.2 mg/kg as reported in Thailand [61]. Arsenic dietary intake primarily derives from fish, shellfish, meat, poultry, dairy products, and cereals. The arsenic found in fish and shellfish typically exists as organic compounds, which are low in toxicity. In regions where arsenic is prevalent in the environment, foods like rice irrigated and cooked with water contaminated with high levels of arsenic significantly contribute to the overall daily arsenic consumption [62]. Rice and fish represent the two main staple foods in Cambodia. Therefore, it is crucial for risk managers to assess the risk of arsenic in food to protect public health and promote fair trade.

For arsenic contamination in fish, according to a subgroup analysis, the analytical method appeared to influence measured arsenic concentrations, with higher values reported in studies using a combination of HPLC/ICP-MS, although this may also reflect co-linearity with publication year or sampling site. There was a strong trend of decreasing arsenic concentrations in more recent studies, which may reflect changes in contamination levels, analytical methods, or study locations. Mean arsenic concentrations varied significantly by province. Kandal had substantially higher pooled levels than Tonle Sap and the other provinces, although internal variability was also high. Previous studies [33,34] indicated a high risk of arsenic contamination in food in Kandal province. The studies also indicated that the likelihood of higher arsenic concentration in Kandal province is a combination of industrialization and naturally occurring phenomena. Arsenic concentrations were significantly higher in farmed and artificial water bodies compared to natural sites (except for one atypically high natural wetland). Burrow pits and controlled environments had the highest mean levels.

In 2015, the FERG of the WHO estimated that more than 1 million illnesses were attributable to four specific metals: arsenic, cadmium, lead, and methyl mercury. Among these, lead was responsible for 54% of the cases, methylmercury accounted for 22%, arsenic contributed to 20%, and cadmium was linked to 1%. Notably, arsenic was the leading cause of mortality, accounting for 96% of deaths, while cadmium was associated with 4% of fatalities [44]. The data presented in Table 5 illustrate the DALYs attributed to elemental toxins in Cambodia, as estimated by the FERG for the Western Pacific Region B. The findings indicate that lead is responsible for the highest incidence of DALYs, with methyl mercury following in significance.

Diet serves as the principal route for exposure to mercury (Hg). Annually, Hg is responsible for approximately 250,000 instances of intellectual disability worldwide, contributing to nearly 2 million disability-adjusted life years (DALYs) on a global scale. Notably, the Western Pacific B subregion is accountable for over one-third of these DALYs [63]. As shown in Table 4, the health risk assessment concerning mercury in POAO indicated that the hazard index was highest for fishery products, with an average mercury index value of 0.75, ranging between 0.5 and 1. The upper range equaled the threshold of 1, underscoring the significant health risks associated with exposure to mercury in fish within Cambodia. It is crucial for the national competent authorities to initiate the environmental monitoring program and to take the necessary measures to prevent and control the mercury contamination in food-producing animals. However, the mercury concentration in other POAOs, such as beef, beef viscera, and egg, was negligible as the values were well below 1.

A study conducted in Indonesia found that consuming a lesser variety of fish significantly increased mercury intake and associated health risks compared to a diversified fish diet, as observed in the Katingan River Basin community. The study also revealed that the average total mercury concentration in fish samples from the Katingan River was quite high (128.67 ± 141.02 µg/kg), exceeding levels reported in similar studies in other countries, including Cambodia and China. The average methylmercury levels in Katingan River fish (15.90 ± 15.96 µg/kg) were also higher than those reported elsewhere, such as Kandal province of Cambodia [64].

Point nine of Article 6 of the Cambodian law on the management of pesticides and fertilizers [65], enacted in 2012, emphasizes the importance of monitoring, regulating, and enforcing legal measures related to all management activities and compliance with standard requirements for pesticides and fertilizers. Likewise, the first point of Article 50 of the law clearly states that the trading of highly toxic pesticides, which are designated as banned in Cambodia or by relevant international treaties, is prohibited.

Our review identified contamination of various POPs in POAO in Cambodia. In terms of PAH contamination, the national competent authority needs to take urgent measures because the BaP and PAH analytical results did not comply with the CAC and the EU, as shown in Table 3. Cambodia is a member of the Stockholm convention on managing POPs. The revision of the National Implementation Plan (NIP) establishes a legislative framework concerning persistent organic pollutants (POPs) in Cambodia. This framework encompasses both international commitments and national regulations pertaining to POPs. It is noteworthy that the majority of legislation addressing POPs in Cambodia does not specifically regulate their management. Within the Cambodian context, the implementation of the Stockholm Convention is facilitated through various regulations related to persistent organic pollutants [19]. A crucial component of the NIP update is the strategy and action plan for the management of POPs in Cambodia. This section delineates the objectives of the NIP, as well as the national priorities and strategies for managing these pollutants.

The majority of fish farms in Cambodia are currently in the process of implementing Good Aquaculture Practices (GAqPs). At present, 32 farms are registered with the Department of Aquaculture Development (DAD), which mandates adherence to fundamental agricultural practices as stipulated by the Aquaculture Order enforced in 2012. Additionally, seven farms have received certification from the DAD in accordance with the Cambodian standards for GAqP. An extensive training initiative has been launched and continues under the CAPFish Aquaculture Program. This program has provided foundational training in GAqP to 2100 farms and households across 10 provinces, with participants attending two to three training sessions. The training activities are set to persist until the conclusion of 2025, aiming to minimize the use of banned substances and to comply with the National GAqP requirements [58].

Project Coordination Unit, 2015, [19] the NRMP’s implementation in Cambodia from 2020 to 2023 utilized a segregated system that focused on 10 out of the 25 provinces. The monitoring efforts focused on three specific species of finfish: pangasius catfish, snakehead, and tilapia. The four NRMP reports from 2020 to 2023 farming seasons we examined indicated the detection of Chloramphenicol, nitrofuran metabolites, and leuco-malachite green, all of which are prohibited substances in food-producing animals in Cambodia and EU member states. This underscores the necessity to broaden the monitoring framework to include additional farmed fish and shrimp species, as well as to conduct further research to gain a comprehensive understanding of antibiotic usage in Cambodia. In a similar vein, antibiotics are extensively employed in Vietnam’s aquaculture sector to manage diseases. Research conducted in Vietnam revealed that the proportion of antibiotics utilized in fish farming exceeded that in shrimp farming. Furthermore, some fish and shrimp farmers in Vietnam have resorted to using banned veterinary medicinal products, indicating a need for mandatory veterinary prescriptions [66].

In terms of other chemical contaminants, except for elemental toxins, POPs, and veterinary drug residues, a study in our review revealed the levels of phthalate esters present in meat and fish. The findings indicated that beef exhibited both the lowest and highest concentrations of phthalate esters, ranging from 0.88 to 2.64 mg/kg. In contrast, the concentration of phthalate esters in clear chicken soup samples analyzed in Thailand was significantly lower, with values between 0.02 and 0.07 mg/kg [67]. We identified an absence of other studies of potential chemical contaminants in POAO that have not yet been publicly available, or very likely, the level of contamination has not yet been assessed in Cambodia.

The WHO estimated that the contamination of aflatoxin in food has the highest DALYs in WPR B, where Cambodia belongs to [3]. However, there has been no known or published study of mycotoxins, particularly aflatoxin, in POAO such as milk, meat, and seafood. The textile and garment sector in Cambodia plays a crucial role in the national economy, contributing significantly to export revenues and providing employment for a substantial number of individuals [68]. Nevertheless, this industry has the potential to produce dioxins, which may lead to the contamination of POAO. Nevertheless, our review did not uncover any prior efforts addressing dioxin, a potential chemical contaminant in POAO.

The studies included in our review provided several recommendations, which emphasized the importance of maintaining ongoing research collaboration. Additionally, they highlighted the necessity of establishing monitoring and surveillance programs, conducting health risk assessments for potential contaminants in widely consumed foods such as fish and vegetables, and enhancing data availability, particularly regarding the daily usage of mercury-containing products in Cambodia.

## 5. Conclusions

This review represents the first systematic literature review and meta-analysis of foodborne hazards in Cambodia, synthesizing available data on arsenic and mercury concentrations in fish. While the number of studies remains limited and many are based on small sample sizes, the analysis offers a structured and transparent approach to aggregating existing evidence. Heterogeneity was high as expected given the diversity of studies (in terms of ecosystem, species, value chain node), small sample sizes, and the challenges of conducting robust surveys and laboratory analyses in low- and middle-income countries (LMICs). In these circumstances, meta-analysis is useful, not just to produce a single estimate, but to characterize the distribution of observed concentrations, assess heterogeneity, and identify data gaps. Cambodia’s newly promulgated Food Safety Law (2022) creates a timely opportunity for evidence-informed decision-making, and this review offers a foundational dataset to guide surveillance, regulatory thresholds, and future research priorities.

In the last 23 years, Cambodia has engaged with both regional and international partners regarding chemical hazards in food and chemical toxins in the marine environment, despite the limited evidence of research collaboration with its neighboring trade partners. It is essential for food control regulators to prioritize bilateral or multilateral partnerships with neighboring countries, particularly Thailand and Vietnam, given the complexities of border food trade, which is often more difficult to regulate due to its informal nature and lower volumes compared to standard trade. The competent authority is advised to focus on conducting similar investigations into biological and chemical toxins in perishable food products, especially products not of animal origin, as well as in drinking water and even animal feed. This approach will facilitate the establishment of a comprehensive risk-based food control system in Cambodia. Furthermore, since previous research on chemical toxins in POAO has predominantly concentrated on fishery products and elemental toxins, it is crucial to broaden the scope to cover sectors beyond fisheries and to include potential contaminants in food with the countrywide geographical coverage. The implementation of a risk-based food control strategy is advised in Cambodia to ensure the optimal use of available resources.

The studies included in this review were solely conducted by a research student, which introduces the potential for selection bias. Furthermore, over the last 23 years, both the equipment utilized and the methodologies employed for laboratory analysis of each toxin have experienced considerable fluctuations. In our review, a significant limitation was the lack of detailed reporting on chemical concentrations. Specifically, only a subset of the included studies provided precise concentration data for muscle and liver tissue in fish samples. Furthermore, a notable omission across many studies was the specification of whether these concentrations were measured on a wet weight or dry weight basis, which complicates the direct comparison of findings. Therefore, the results of the review should be interpreted with caution. Nevertheless, the results offer valuable insights that serve as a solid foundation for future work. This review also provides foundational evidence to support the development of a more robust National Food Control System (NFCS). The use of systematic reviews and meta-analyses, as demonstrated in this study, should be institutionalized as part of Cambodia’s national risk analysis framework to guide decision-making, prioritize resources, and protect public health.

## Figures and Tables

**Table 1:** List of certain carcinogenic chemicals that can be found in products of animal origin.

Name of Carcinogenic Chemical Hazard	Food Source (Products of Animal Origin)	High Risk Group	Reference
Heavy metals			
Arsenic (As)—Inorganic form	Fish, shellfish (especially near industrial zones)	YOPI group (Young, Old, Pregnant, Immunocompromised)	[11]
Cadmium (Cd)	Liver, kidney (offal), shellfish (mussels, oysters)	YOPI, especially fetuses and young children	[12]
Lead (Pb)	Meat (game hunted with lead bullets), canned fish	YOPI, especially young children	[13]
Methyl mercury (MeHg)	Fish	YOPI, especially pregnant women and young children	[14]
Persistent organic pollutants (POPs)			
Dioxins	Fish, meat, and dairy products	Especially young children and infants	[15]
Polychlorinated Biphenyls (PCBs)	Fish, meat, dairy products	Young children and individuals with high consumption of fatty POAO	[16]
DDT & Metabolites	Fish and meat	YOPI	[17]
Polycyclic aromatic hydrocarbons (PAHs)			
Benzo[a]pyrene (BaP)	Grilled and smoked fish and meat	YOPI	[18]
Dibenzo[a,h]anthracene	Processed meat and smoked cheese	YOPI	[18]
Benz[a]anthracene	Grilled and smoked fish and meat	YOPI	[18]

**Table 2:** Quality assessment criteria for full-text review.

Quality Criteria	Good	Medium	Poor
Scientific method	-Clarity of study, including subject, setting, and sampling points in detail -Clear description of appropriate sampling method	-Detailed description of study subject, setting, and sampling points, but somewhat unclear. -Sampling methods were not clearly described but contained enough information for data extraction	-The key part of study setting for example sampling points are not described at all. -Unclear/invalid sampling methods
Testing method	-Standard laboratory testing methods were used (use of relevant ISO method or equivalent), and accuracy of equipment meets the needs of the national/EU/Codex	-The laboratory testing methods were acceptable or valid (use of the AOAC method or equivalent), and accuracy of equipment meets the needs of the national/EU/Codex	-The laboratory testing method was not acceptable or was invalid (use of rapid test kit). Use of equipment did not guarantee the accuracy of results
Information for use of equipment	-Detailed information available for use of equipment	-Sufficient information for use of equipment	-Insufficient or unclear information for use of equipment
Results accuracy for data extraction	-Detailed and accurate results	-Sufficient results for data extraction	-Insufficient or incomplete results presentation

**Table 3:** Summary of evidence of chemical hazards in products of animal origin in Cambodia from 2000 to 2023, reported by 23 studies included in the review.

Name of Hazard	Name of Food/Source	Sampling Location	Sampling Point	Total Number of Samples	Test Methods	Equipment Used	Ref. Paper No. (See S1)
Arsenic (Inorganic arsenic and total arsenic)	Snails, clams, fish, meat, viscera	Kampong Cham, Kandal, Kratie, Tonle Sap	Aquaculture site and nature (lakes, ponds, rivers)	331	Acid digestion, internal procedures	HPLC-ICP-MS, ICP-MS, ICP-OES, ETAAS	1, 2, 10, 14, 15, 16, 18
Benzo[a]pyrene	Processed fishery products	Battambang, Kampong Cham, Kampong Chhnang, Kandal, Siem Reap	Processing sites and markets	105	Accelerated solvent extraction, modified QuECheRS	GC/MS, HPLC-FLD	5, 11, 13
Cadmium	Fish and fishery products	Tonle Sap, Phnom Penh	Coastal water	208	Acid digestion method	ICP-MS, ETAAS	9, 10, 14, 18
Chloramphenicol	Freshwater farmed fish	Kandal, Prey Veng, Takeo, Kampong Cham, Kampong Chhnang, Pursat, Battambang, Kampong Thom, Siem Reap, and Banteay Meanchey	Freshwater fish farms	129	LC/MS/MS	LC-MS/MS	FiA report 1, 2, 3, 4
Hexachlorobenzene (HCB)	Fish, mussels, meat, viscera	Kampong Cham, Kratie, Kandal, Sihanoukville	Coastal water and markets	184	USEPA 3620B and 3640A, lipid extraction	GC-MSD, GC-ECD	3 (46 samples were collected not only from various locations in Cambodia but from China, Hong Kong, India, Indonesia, Japan, Malaysia, the Philippines, and Vietnam), 17
hexachlorocyclohexane isomers (HCHs)	Fish, meat, viscera	Kampong Cham, Kratie, Kandal	Coastal water	46	Lipid extraction	GC-ECD	3
Lead	Fish	Coastal areas, Phnom Penh, Tonle Sap, Kandal, Prey Veng, Takeo, Kampong Cham, Kampong Chhnang, Pursat, Battambang, Kampong Thom, Siem Reap, and Banteay Meanchey	Coastal water, lake and freshwater fish farms	249	Acid digestion	ICP-MS	9, 10, 14, 18, and FiA report 1, 2 & 3
Mercury	Fish, meat, viscera, egg	Kandal, Prey Veng, Takeo, Kampong Cham, Kampong Chhnang, Pursat, Battambang, Kampong Thom, Siem Reap, and Banteay Meanchey Phnom Penh, Tonle Sap	Coastal water, farms, and markets	495	Acid digestion, USEPA 7473, standard addition method,	AAS, Direct mercury analyser, ICP-MS	7, 9, 10, 12, 19, and FiA report 1, 2,3,4
∑Organochlorine pesticides (∑Organochlorine pesticides included the summation of DDT, HCH, CHL, DRINs, Mirex, and HCB)	Mussels, fish, meat, viscera	Kampong Cham, Kandal, Kratie	Markets	138	USEPA 3620B and 3640A	GC-MSD	17
Phthalate esters	Fish, meat, viscera	Kampong Cham, Kandal, Kratie	Farms and markets	42	USEPA 3540C and 3620B	GC-MS	6
Polycyclic aromatic hydrocarbon (PAH4)	Smoked freshwater fish	Battambang, Kampong Cham, Kampong Chhanag, Kampong Thom, Kandal, Siem Reap	Market, processing sites	105	In-house method, Accelerated solvent extraction, modified QuECheRS	HPLC-FLD, GC-MS	5,11,13
Polycyclic aromatic hydrocarbon (PAH12)	Mussels, smoked freshwater fish	Battambang, Kampong Cham, Kampong Chhnang, Kandal, Koh Kong	Jetty, wharf wall, buoys, and rocks, processing sites	>87 (>30 + 57)	In-house extraction method, modified QuECheRS	GC-MS	8 (Paper 8 did not specify the exact number of samples; instead, it stated that more than 30 samples were collected), 11
polybrominated diphenyl ethers (PBDEs)	Green mussels and blue mussels	Koh Kong, Koh Preab, Sihanoukville	Coastal water	46	Lipid extraction	GC-ECD	3
Total bytyltin	Green mussels	Koh Kong, Sihanoukville	Fisheries sites and international and commercial harbors	419	Modified lipid extraction	GC-FPD	4
Tri butyltin	Green mussels	Koh Kong, Sihanoukville	Fisheries sites and international and commercial harbors	419	Modified lipid extraction	GC-FPD	4

**Table 4:** Evidence of concentration of chemical contaminants in different POAO in Cambodia from 2000 to 2023.

Chemical Contaminant	Product	Minimum Concentration in mg/kg	Maximum Concentration in mg/kg	Average Concentration in mg/kg	% Exceeded National MRL (Mostly Based on Codex’s MRL)	% Exceeded the EU’s MRL/Action Level
Arsenic	Beef	0.004	0.1	0.056	NA	NA
Arsenic	Beef viscera	0.002	0.03	0.01	NA	NA
Arsenic	Egg	0.02	0.06	0.04	NA	NA
Arsenic	Pork	0.01	0.02	0.015	NA	NA
Benzo[a]pyrene	Fishery products	0.005	0.9	0.13	94.29	100
Cadmium	Fish	0.0003	0.28	0.035	9.09	9.09
Chloramphenicol	Freshwater fish	0.03	1.18	0.255	100	100
Lead	Freshwater fish	<LOD	0.31	0.05	5.88	5.88
Mercury	Beef	0.004	0.02	0.01	NA	NA
Mercury	Beef viscera	0.001	0.01	0.004	NA	NA
Mercury	Egg	0.001	0.024	0.02	NA	NA
Mercury	Pork	0.002	0.02	0.01	NA	NA
∑Organochlorine pesticides	Beef	0.01	0.04	0.025	100	100
∑Organochlorine pesticides	Beef viscera	0.007	0.19	0.05	100	100
∑Organochlorine pesticides	Egg	0.009	0.028	0.018	100	100
∑Organochlorine pesticides	Fishery products	0.008	0.04	0.019	100	100
∑Organochlorine pesticides	Pork	0.009	0.14	0.01	100	100
Phthalate esters	Beef	0.88	2.65	1.73	NA	NA
Phthalate esters	Beef viscera	0.99	2.28	1.87	NA	NA
Phthalate esters	Fishery products	0.92	2.26	1.59	NA	NA
Phthalate esters	Pork	0.92	1.24	1.08	NA	NA
Polycyclic aromatic hydrocarbon (PAH 4)	Processed fishery products	0.034	17.2	1.92	NA	100
Polycyclic aromatic hydrocarbon (PAH 12)	Mussels, processed fishery products	0.021	17.2	4.05	NA	NA
Tributyltin (Wet weight)	Marine fish	0.0024	0.088	0.015	NA	NA
Total butyl tin (wet weight)	Marine fish	0.0024	0.15	0.027	NA	NA

**Table 5:** Evidence of PBDE and organochlorine pesticides in marine fish and mussels.

Product	Hazard	Location	Concentration in mg/kg	Reference (See S1)
Mussels	PBDEs (mono-hepta)	Sihanoukville	0.0053	Paper 3
Mussels	PBDEs (mono-hepta)	Sihanoukville	0.0023	
Mussels	HCB	Sihanoukville	<0.001	
Mussels	HCB	Sihanoukville	<0.001	
Fish	HCB	Kampong Cham	0.00009	Paper 17
Fish	HCB	Kratie	0.00025	
Fish	HCB	Kandal	0.00107	
Fish	CHLs	Kampong Cham	0.00035	Paper 17
Fish	CHLs	Kratie	0.00111	
Fish	CHLs	Kandal	0.0032	
Fish	DRINs (DRINs is the combination of aldrin, dieldrin, and endrin)	Kampong Cham	0.00051	Paper 17
Fish	DRINs	Kratie	0.00065	
Fish	DRINs	Kandal	0.00294	
Fish	Mirex	Kampong Cham	0.00045	Paper 17
Fish	Mirex	Kratie	0.00031	
Fish	Mirex	Kandal	0.00035	
Mussels	DDTs	Sihanoukville	0.12	Paper 3
Mussels	DDTs	Sihanoukville	0.12	
Fish	DDTs	Kampong Cham	0.00547	Paper 17
Fish	DDTs	Kratie	0.00949	
Fish	DDTs	Kandal	0.023	
Mussels	PCBs	Sihanoukville	0.3	Paper 3
Mussels	PCBs	Sihanoukville	0.2	
Mussels	CHLs	Sihanoukville	0.0041	Paper 3
Mussels	CHLs	Sihanoukville	0.0025	
Mussels	HCHs	Sihanoukville	0.0055	Paper 3
Mussels	HCHs	Sihanoukville	0.0072	
Fish	HCHs	Kampong Cham	0.00101	Paper 17
Fish	HCHs	Kratie	0.00186	
Fish	HCHs	Kandal	0.00572	

**Table 6:** Health risk assessment of exposure to cadmium and mercury contamination in POAO in Cambodia.

Hazard	Name of Product	HI (Minimum)	HI (Maximum)	HI (Average)
Cadmium	Freshwater fish	0.002	0.28	0.22
Mercury	Beef	0.002	0.008	0.004
Mercury	Beef viscera	0.0004	0.004	0.002
Mercury	Egg	0.0004	0.01	0.008
Mercury	Fish	0.5	1.0	0.75
Mercury	Pork	0.002	0.01	0.008

## Data Availability

Data are contained within the article and Supplementary Materials.

## Supplementary Materials

The following supporting information can be downloaded at: https://www.mdpi.com/article/10.3390/ijerph22081299/s1, S1: List of published papers and reports included in the review, S2: Meta-analysis for arsenic in fish, S3: Meta-analysis for mercury in fish.

## Abbreviations

The following abbreviations are used in this manuscript:

ADI	Acceptable daily intake
AFRO	Africa Regional Office
AMRO	America Regional Office
AOAC	Association of Official Agricultural Chemists
BaP	Benzo[a]pyrene
BW	Body weight
C	Concentration of the contaminant
CAC	Codex Alimentarius Commission
CCS	Cambodian Chemical Society
CHLs	Heptachlor, trans-chlordane, cis-chlordane, trans-nonachlor, and cis-nonachlor
CKD	Chronic kidney disease
CTTF	Chemicals and Toxins Task Force
DAD	Department of Aquaculture Department
DALYs	Disability Adjusted Life Years
DDT	dichloro-diphenyl-trichloroethane
DG SANTE	Directorate-General for Health and Food Safety
DL	DerSimonian–Laird method
DRINs	Aldrin, dieldrin, and endrin
EDI	Estimated daily intake
EFSA	European Food Safety Authority
EMRO	Eastern Mediterranean Regional Office
ETAAS	Electrothermal atomic absorption spectrometry
EU	The European Union
EURO	European Regional Office
EWI	Estimated weekly intake
F	Weekly food consumption per person
FAO	Food and Agriculture Organization of the United Nations
FERG	Foodborne Disease Burden Epidemiology Reference Group
FiA	Fisheries Administration
FLD	Fluorescence detector
GAqP	Good aquaculture practices
GC	Gas chromatography
GDAHP	General Directorate of Animal Health and Production
HCB	Hexachlorobenzene
HCH	Hexachlorocyclohexane
HI	Hazard index
HPLC	High-performance liquid chromatography
ICP-MS	Inductively coupled plasma mass spectrometry
ICP-OES	Inductively coupled plasma optical emission spectroscopy
ID	Intellectual disability
ILRI	International Livestock Research Institute
JECFA	The Joint FAO/WHO Expert Committee on Food Additives
kg	kilogram
LC-MS	Liquid chromatography-mass spectrometry
LOD	Limit of detection
LOQ	Limit of quantification
MAFF	Ministry of Agriculture, Forestry and Fisheries
Mirex	Organochlorine insecticide
MS	Mass spectrometry
NFCS	National Food Control System
NRMP	National residue monitoring plan
NIP	National implementation plan
OCP	Organochlorine pesticide
PAH	Polycyclic aromatic hydrocarbon
PBDE	Polybrominated Diphenyl Ethers
PCB	Polychlorinated biphenyls
PFOS	Perfluorooctane sulfonate
POAO	Products of animal origin
POPs	Persistent organic pollutants
PRISMA	Preferred Reporting Items for Systematic Review and Meta-analysis
PROSPERO	International database for registering systematic review protocols
QuEChERS	Quick, Easy, Cheap, Effective, Rugged, and Safe
REML	Restricted Maximum Likelihood
SEARO	Southeast Asia Regional Office
SLR	Systematic literature review
TWI	Total weekly intake
UNEP	United Nations Environment Programme
UNIDO	United Nations Industrial Development Organization
USAID	United States Agency for International Development
USEPA	United States Environmental Protection Agency
VMP	Veterinary medical products
W	Mean body weight
WHO	World Health Organization
WPR B	Western Pacific Region B
WPRO	Western Pacific Regional Office
