# Supplementary material for: Chemical Hazards in Products of Animal Origin in Cambodia from 2000 to 2023: A Systematic Review and Meta-Analysis
Source: Int J Environ Res Public Health. 2025 Aug 19;22(8):1299. doi: 10.3390/ijerph22081299 (PMC12386664; doi:10.3390/ijerph22081299)
Supplement: Supplementary file 1 [file ijerph-22-01299-s001.zip › Supplementary material S1.pdf]

Systematic literature review of hazards in fish in Cambodia – meta-analysis
Delia Grace draft v1 21st April

Methods

We conducted a random-effects meta-analysis, which assumes that each study estimates a different true effect and that these effects are distributed around an overall average. This model accounts for both within-study sampling error and between-study heterogeneity, making it appropriate when substantial heterogeneity is expected.

The between-study variance (τ^2) was estimated using Restricted Maximum Likelihood (REML), which is more robust than the traditional DerSimonian–Laird (DL) method, particularly when heterogeneity is high. To improve the accuracy of standard errors and confidence intervals, we applied the Knapp–Hartung adjustment, which is recommended in random-effects models with either few studies or high heterogeneity.

Initial data exploration showed that the distribution of mean arsenic concentrations was strongly right-skewed and leptokurtic. To improve normality, we applied a log transformation. After transformation, the distribution did not deviate significantly from normality ($p = 0.675$, Stata ladder test).

All analyses were conducted on the log-transformed means. We conducted sensitivity meta-analyses using alternative estimators (e.g., DL, Paule–Mandel) to assess the robustness of the pooled effect.

Subgroup analyses were conducted using restricted maximum likelihood (REML) estimation in Stata, with Cochran's Q used to test for heterogeneity between subgroups. Subgroup means are presented as geometric means (back-transformed from log-transformed data), with 95% confidence intervals and chi-squared tests of between-group differences.

Sub-group analysis guided the selection of a parsimonious meta-regression model by identifying the most influential and non-collinear predictors, thereby reducing the risk of multicollinearity and overfitting in the regression analysis. Meta-regression was performed using random-effects models to explore potential sources of heterogeneity. Standard model diagnostics included checks for residual normality, outliers, and influential studies.

Lastly, potential publication bias and small-study effects were assessed using funnel plots, trim and fit analysis and the Egger test

Results and discussion

1. Pooled estimate

Across 43 studies, the pooled geometric mean arsenic concentration in fish was 0.40 µg/g (95% CI: 0.25–0.66 µg/g). This was statistically significantly lower than 1 µg/g ($t(42) = -3.71$, $p = 0.0006$), as confirmed by the test of the pooled log-transformed effect size.

The 95% prediction interval ranged from 0.015 to 10.50 µg/g, indicating that a future study under similar conditions could plausibly report arsenic concentrations anywhere in this range — consistent with extensive between-study heterogeneity.

Heterogeneity statistics supported this ($Tau^2 = 2.55$, $I^2 = 99.99\%$, Cochran's Q = 210,000 (df = 42, $p < 0.0001$), H^2 = 11,230.) These results confirm extreme between-study variability, beyond what would be expected by chance alone.

2. Forest Plot

The forest plot of the log-transformed mean arsenic concentrations is presented in Figure 1, showing the individual study estimates with confidence intervals, and the pooled effect size. All studies received similar weights due to the dominance of between-study variance (τ^2) over within-study sampling error.

Figure 1. Forest plot of mean arsenic concentration in fish (log scale), Cambodia.

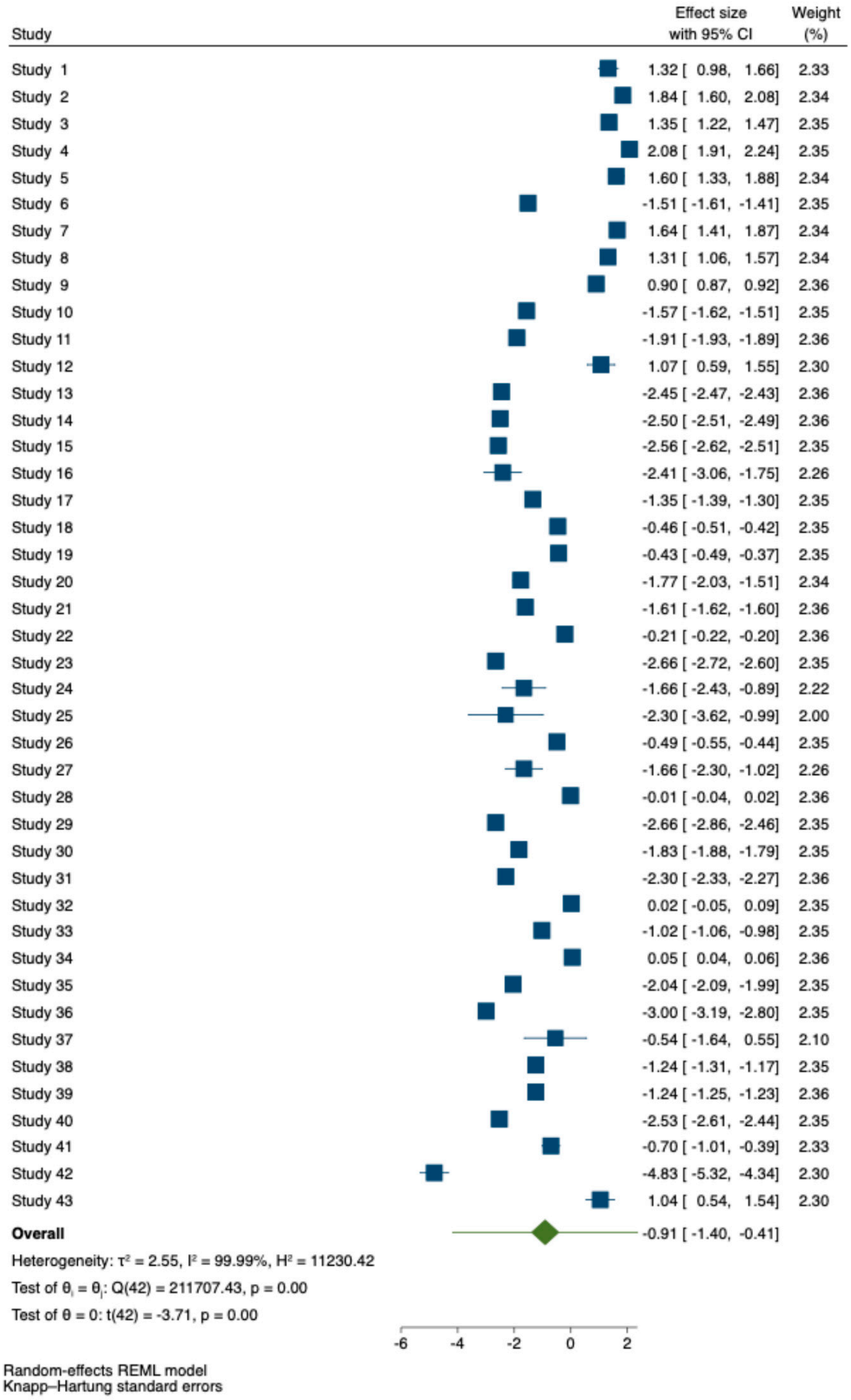

3. Sub-group analysis and meta-regression

Sub-group analysis was conducting using REML sub-group meta-analysis in Stata and Cochran's Q to test heterogeneity across sub-groups.

Analytical method appeared to influence measured arsenic concentrations, with higher values reported in studies using HPLC/ICP-MS, although this may also reflect co-linearity with publication year or sampling site.

There was a strong trend of decreasing arsenic concentrations in more recent studies, which may reflect changes in contamination levels, analytical methods, or study locations.

Mean arsenic concentrations varied significantly by province. Kandal had substantially higher pooled levels than Tonle Sap and the other provinces, although internal variability was also high.

Arsenic concentrations were significantly higher in farmed and artificial water bodies compared to natural sites (with the exception of one atypically high natural wetland). Burrow pits and controlled environments had the highest mean levels

We fitted a parsimonious meta-regression model informed by subgroup analysis, excluding one extreme outlier (a natural wetland). The model explained 37% of the between-study variance (R^2 = 37.1%) and significantly improved model fit (Wald $\chi^2(4)$ = 27.50, $p < 0.001$). Arsenic concentrations were substantially higher in studies sampling from artificial environments, with a borderline significant coefficient (β = 2.57, 95% CI: –0.05 to 5.18, p = 0.054), corresponding to an estimated 13-fold increase compared to natural sites. Other predictors, including publication year and analytical method, were not independently associated with mean arsenic levels after adjusting for site type. Residual heterogeneity remained high (τ^2 = 1.52, I^2 = 99.98%), indicating that additional unmeasured factors may contribute to variability.

Meta-regression univariable

Predictor	**Coefficient (β)**	**p-value**	**R^2 (%)**	**Interpretation (Back-transformed if relevant)**	Include in model
Paper id (**paperno**)	–0.33	<0.001	20.9%	Later-numbered papers report lower arsenic. A random effect or grouping variable	No RE
Sample size (**samplesize**)	–0.17	0.001	0.0%	Larger studies report lower arsenic. Explains 0% - probably a confounder	No confounder
Years ago published (**publishedyearago**)	–0.059	0.040	0.0%	More recent studies report higher arsenic	Small effect 0 r2
Fish sampled (**fish**)	+1.38	0.009	19.3%	Arsenic ~**4.0× higher** in fish vs. other aquatic animals (exp(1.38))	Yes
Artificial site (**artificial**)	+2.45	<0.001	35.4%	Arsenic ~**11.6× higher** in artificial sites (exp(2.45))	Yes
Method: ICP-MS (**icp_ms**)	–1.86	<0.001	25.0%	Arsenic ~**6.4× lower** than reference (exp(–1.86))	No redundant
Method: HPLC/ICP-MS (**hplcicpms**)	+2.59	<0.001	40.0%	Arsenic ~**13.3× higher** (exp(2.59))	yes
Method: ICP-OES (**icp_oes**)	–0.63	0.517	0.0%	Not significant	No redundant
Province: Tonle Sap (**tonlesap**)	–1.19	0.011	11.7%	Arsenic ~**70% lower** (exp(–1.19) ≈ 0.30×)	yes
Province: Kandal (**kandal**)	+2.19	<0.001	37.4%	Arsenic ~**8.9× higher** (exp(2.19))	yes
Province: Kratie (**kratie**)	–1.25	0.189	1.8%	Not significant	No
Province: Kampong Cham (**kampong**)	–1.10	0.250	0.8%	Not significant	

Meta-regression multivariable

Although no individual predictor was statistically significant in the multivariable model, the model as a whole explains ~44% of the heterogeneity, and the effect sizes remain large and directionally consistent with univariable findings. This suggests that multiple factors jointly influence arsenic concentrations, and there is substantial confounding or shared variance between predictors (e.g., certain methods used only at certain sites

4. Publication bias and small study effect

We assessed publication bias using the trim-and-fill method and the Egger regression test. The trim-and-fill procedure did not impute any missing studies, and the pooled effect estimate remained unchanged, suggesting no evidence of publication bias. Similarly, the Egger test indicated no small-study effects (β = –0.36, p = 0.83), further supporting the robustness of the results.

Visual inspection of the funnel plot indicated no strong asymmetry, supporting the Egger test and trim-and-fill results which did not suggest publication bias. However, the funnel was widely dispersed, consistent with the very high heterogeneity observed (I^2 = 99.98%). This heterogeneity likely reflects real differences between studies in terms of sampling site, species, and analytical methods.

Figure 2: Funnel Plot and bias assessment

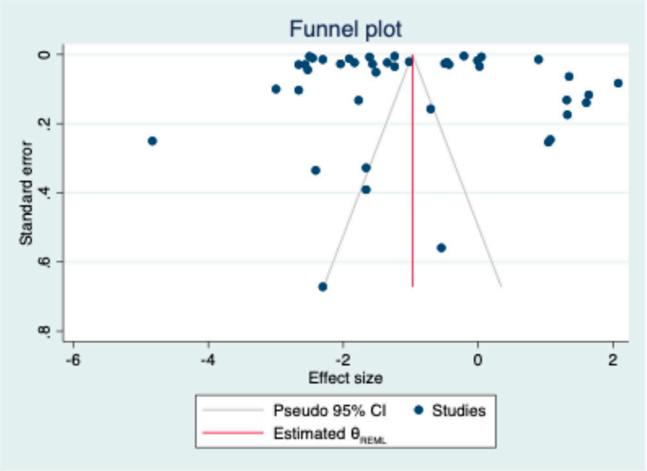

```
Stata code and output and explanation
```

```
import excel using "arsenic.xlsx", firstrow clear
```

1. Getting sample size, mean concentration and SD

For 26 we have mean and sd and for 19 we have mean and range .
According to (Weir et al., 2018) the recommended method for calculating the s.d. when range is given is that of (Walter & Yao, 2007). There are several methods to calculate sd from range. The most simple is the formula SD=RANGE/4 (Hozo et al., 2005) . This is good for small samples. (Walter & Yao, 2007) provide an improved approach calculated the SD as (upper bound – lower bound)/ conversion factor based on sample size. The conversion factor is provided in look up tables and a formula. This works better for larger samples or skewed data.

In our case we have 19 and sample size is <12. When n<15 the method of Hozo et al works as well or better than the more sophisticated Walter & Yao

2. Generate standard error for ach study

SD is a measure of how spread out a normal distribution is. 68% of observations are within 1 sd, 95 within in 2 sd and 99.7 within 3 sd. If you take lots of sample from a population, their means will follow a normal curve. The SD of this normal curve is the SE. Standard error helps estimate how well your sample data represents the whole population. High SE means data widely spread and not representative. Decrease SE by increasing sample size

```
. gen se= sd/sqrt(samplesize)
```

3. Check that all SE are >0

```
standard-error variable se must be positive r(459);
```

Two studies have only two samples and the upper bound and lower bound and mean are the same. So no variation between the samples so SD = 0 so SE = 0. Meta-regression maths can't work with se=0

Also, if n=2, You can't estimate a meaningful SD or SE. Adds almost no information but can bias results (especially if variance is zero — inflates precision unfairly). For now, we will drop the two Lake et al studies with se=0

4. Check normality of mean concentration

- The standard random-effects meta-analysis assumes approximately normally distributed effect sizes.
- In random-effects models, weights are based on both within-study variance (SE^2) and between-study variance (τ^2). A few large values (like the ones near 7–8 µg/g) can drive apparent between-study variation. This can lead to extremely high τ^2 and I^2 (proportion variability due to real difference and not sampling)

```
histogram meanconcentration, width(0.5) frequency normal
```

width(0.5) controls the bin width — adjust as needed.
normal overlays a normal curve for comparison.

```
qnorm meanconcentration
```

This directly tests normality by comparing quantiles of your data with a normal distribution:
*If the points **deviate from the straight line**, the data are not normally distributed.*

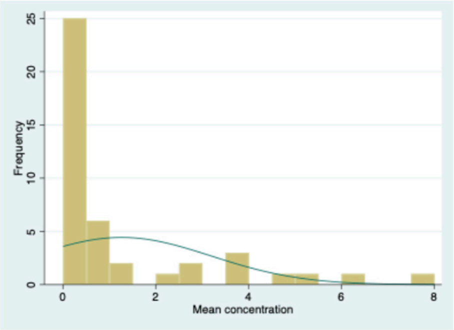

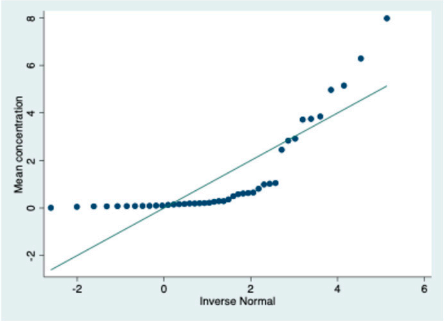


```
. summarize meanconcentration, detail

                        Mean concentration
-------------------------------------------------------------
      Percentiles      Smallest
 1%         .008           .008
```

```
 5%          .07            .05
10%         .077            .07       Obs                  43
25%           .1            .07       Sum of wgt.          43

50%          .29                      Mean           1.264535
                        Largest       Std. dev.      1.934964
75%         1.05           4.97
90%         3.85           5.15       Variance       3.744085
95%         5.15           6.29       Skewness       1.866843
99%         7.98           7.98       Kurtosis       5.672381
```

SD>mean. A lot of variation and right skew.
Skew +2 = big, positive (rightwards) skew (0=symmetric, 1 moderate skew, 2 strong skew)
Kurtosis 6 = fat, leptokurtic tails

```
. ladder meanconcentration

Transformation         Formula               chi2(2)   Prob > chi2
------------------------------------------------------------------
Cubic                  meanco~n^3              48.55         0.000
Square                 meanco~n^2              36.95         0.000
Identity               meanco~n                18.69         0.000
Square root            sqrt(meanco~n)           8.71         0.013
Log                    log(meanco~n)            0.79         0.675
1/(Square root)        1/sqrt(meanco~n)        35.98         0.000
Inverse                1/meanco~n              60.29         0.000
1/Square               1/(meanco~n^2)          64.67         0.000
1/Cubic                1/(meanco~n^3)          64.75         0.000
```

Null hypothesis: The transformed variable is normally distributed.
High p-value (close to 1): We fail to reject normality → transformation brings us close to normal

5. Log transform concentration and se

Log transformation can
- Pull in the long right tail (reduces skew)
- Stabilize the variance
- Make your data more normal-like (closer to what meta-analysis assumes)
- Prevent negative predictions (because log-transformed values are unbounded below, but back-transformed values are always > 0)

The pooled theta is now the **log of the geometric mean** arsenic concentration.
You can interpret results in µg/g by applying: "display exp(theta)" and "display exp([95% CI bounds])

generate logmean = log(meanconcentration)
generate logse = se / meanconcentration
generates approximate standard error of the log-transformed mean, using the delta method.

(Delta method formula
Often in addition to reporting parameters fit by a model, we need to report some transformation of these parameters. The transformation can generate the point estimates of our desired values, but the standard errors of these point estimates are not so easily calculated. They can, however, be well approximated using the delta method. The delta method approximates the standard errors of transformations of random variable using a first-order Taylor approximation.)

. hist logmean, width(0.5) frequency normal
(bin=14, start=-4.8283138, width=.5)

. qnorm logmean

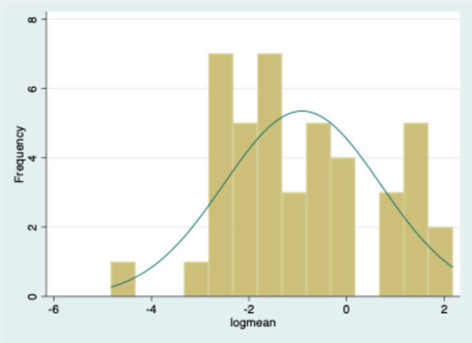

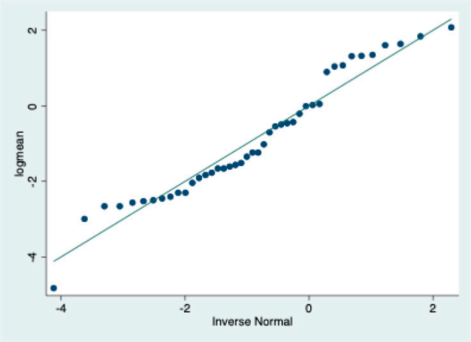

6. Set as meta regression in Stata

Mean concentration is stored as a string so need to first de-string

. describe meanconcentration
. destring meanconcentration, replace force

Use the third option “Declare generic precomputed effect sizes” and set with meanconcentration effect size and standard error SE

```
meta set logmean logse
```

7. Conduct meta-regression in stata

Set Model

- Random effects assume there is real difference across studies not just sampling error. This is likely the case as the studies come from different environments and fish.
- Fixed effects- assumes one underlying correct true effect. Common effect- same as fixed with inverse-variance weighting

Set Method

Method	Description	Recommendation
**REML**	Restricted Maximum Likelihood — robust and widely recommended	Best all-rounder
ML	Maximum Likelihood — like REML but may underestimate τ^2	Avoid unless ML-based model comparison is needed
DerSimonian-Laird (DL)	Classic method, fast but less accurate when heterogeneity is high	Use only for legacy comparison
Hedges	Unbiased but can be unstable for small numbers of studies	Not needed here
Paule-Mandel PM	Performs well in many settings, alternative to REML	Reasonable backup
Hunter-Schmidt	Used in psychometrics, not ideal here	Not suitable
Sidik-Jonkman	Overestimates τ^2 when heterogeneity is low	Too conservative

Select RE options

- Sensitivity meta-analysis: compares REML to alternative tau2 methods like DL or PM. Hels see if pooled estimate robust across methos
- Report prediction interval: gives a 95% prediction interval (PI) where the effect size in a *new* study is likely to fall. CIs tell you the precision of your estimate of the *mean*. PIs tell you how much the effect might vary in a new setting — very useful for generalizability
- Standard error adjustment for ES. Adjusts se when very few studies, very unequal sample sizes, or heteroskedasticity – probably not needed

```
. meta summarize, random(reml) predinterval(95) se(khartung)

  Effect-size label: Effect size
```

```
        Effect size: logmean
          Std. err.: logse

Meta-analysis summary                     Number of studies =     43
Random-effects model                      Heterogeneity:
Method: REML                                           tau2 =  2.5452
SE adjustment: Knapp-Hartung                         I2 (%) =   99.99
                                                         H2 = 11230.42
```

- *Tau or between study variance (true heterogeneity between studies) is 2.5 which is large but less than 3 which was the tau2 of the untransformed data*
- *12% is the proportion of variability due to real differences rather than chance or sampling error and was 99.99% which was very large*
- *H2 is another heterogeneity metric where 1 means perfect heterogeneity. 11,230 means the observed variability is over 11,000 times higher than what you'd expect if there were no heterogeneity.*
- *Knapp–Hartung adjustment refines the standard errors and confidence intervals to account for uncertainty in tau^2. It is recommended for random-effects models with fewer studies or high heterogeneity, as in this case.*

Study	Effect size	[95% conf.	interval]	% weight
Study 1	1.322	0.980	1.663	2.33
Study 2	1.839	1.602	2.076	2.34
Study 3	1.348	1.223	1.473	2.35
Study 4	2.077	1.915	2.239	2.35
Study 5	1.603	1.330	1.877	2.34
Study 6	-1.514	-1.615	-1.413	2.35
Study 7	1.639	1.410	1.868	2.34
Study 8	1.314	1.056	1.572	2.34
Study 9	0.896	0.868	0.924	2.36
Study 10	-1.565	-1.620	-1.511	2.35
Study 11	-1.911	-1.934	-1.887	2.36
Study 12	1.071	0.589	1.553	2.30
Study 13	-2.453	-2.474	-2.433	2.36
Study 14	-2.501	-2.512	-2.490	2.36
Study 15	-2.564	-2.621	-2.507	2.35
Study 16	-2.408	-3.065	-1.751	2.26
Study 17	-1.347	-1.393	-1.301	2.35
Study 18	-0.462	-0.506	-0.418	2.35
Study 19	-0.431	-0.487	-0.374	2.35
Study 20	-1.772	-2.031	-1.513	2.34
Study 21	-1.609	-1.624	-1.595	2.36
Study 22	-0.211	-0.219	-0.202	2.36
Study 23	-2.659	-2.716	-2.602	2.35
Study 24	-1.661	-2.427	-0.895	2.22
Study 25	-2.303	-3.619	-0.986	2.00
Study 26	-0.494	-0.545	-0.443	2.35
Study 27	-1.661	-2.303	-1.019	2.26
Study 28	-0.010	-0.044	0.024	2.36
Study 29	-2.659	-2.861	-2.457	2.35
Study 30	-1.833	-1.879	-1.786	2.35
Study 31	-2.303	-2.331	-2.274	2.36
Study 32	0.020	-0.047	0.087	2.35
Study 33	-1.022	-1.062	-0.981	2.35
Study 34	0.049	0.036	0.062	2.36
Study 35	-2.040	-2.094	-1.987	2.35
Study 36	-2.996	-3.192	-2.800	2.35
Study 37	-0.545	-1.640	0.551	2.10
Study 38	-1.238	-1.306	-1.170	2.35
Study 39	-1.238	-1.248	-1.228	2.36
Study 40	-2.526	-2.612	-2.439	2.35
Study 41	-0.703	-1.012	-0.395	2.33
Study 42	-4.828	-5.318	-4.338	2.30
Study 43	1.041	0.544	1.538	2.30
theta	-0.907	-1.401	-0.414	

```
95% prediction interval for theta: [-4.167, 2.352]

Test of theta = 0: t(42) = -3.71              Prob > |t| = 0.0006
Test of homogeneity: Q = chi2(42) =  2.1e+05    Prob > Q = 0.0000
```

Exp(theta)= exp(-0.907) = 0.403 µg/g

```
display exp(-0.907)
.40373361
```

exp(−1.401)≈0.246 µg/g,exp(−0.414)≈0.661 µg/g

0.403 (0.246-0.661 µg/g)

The pooled average arsenic concentration in fish is approximately 0.40 µg/g, with a 95% CI of 0.25 to 0.66 µg/g.

```
exp(−4.167)≈0.015 µg/g,exp(2.352)≈10.50 µg/g
95% prediction interval for theta: 0.015 - 10.50 µg/g
```

In a new study under similar conditions, the mean arsenic concentration could plausibly fall anywhere between 0.015 and 10.5 µg/g

Weights are very similar partly because sample size were similar. But in random-effects models, weights are based on both within-study variance (SE^2) and between-study variance (tau^2). When tau^2 is large, it dominates the weighting scheme, and all studies contribute nearly equally, regardless of their individual precision. That's why studies with low SE still get similar weights to those with higher SE.

```
Test of theta = 0: t(42) = -3.71                Prob > |t| = 0.0006
```

(Since the pooled log-mean is negative, this translates to a pooled concentration < 1 µg/g. the pooled log-mean is significantly different from 0 (t(42) = –3.71, p = 0.0006), confirming that average arsenic concentration is significantly lower than 1 µg/g.

```
Test of homogeneity: Q = chi2(42) =  2.1e+05       Prob > Q = 0.0000
```

(confirms massive heterogeneity; as vastly greater than the df 42 and highly significant)

8. Conduct Forest plot

meta forestplot, random(reml) predinterval(95) se(khartung)

. meta galbraith, random(reml)

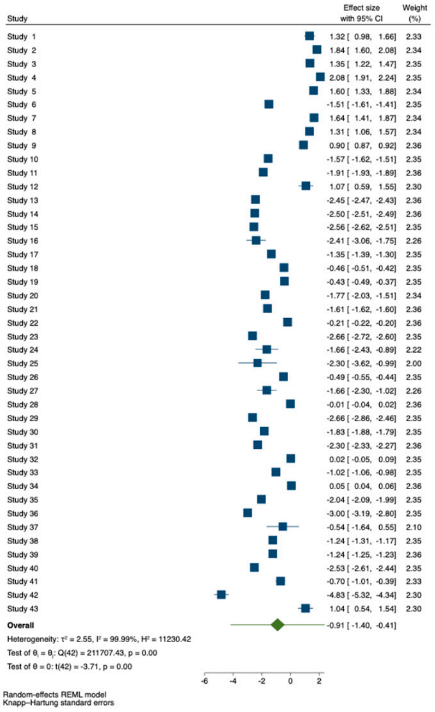

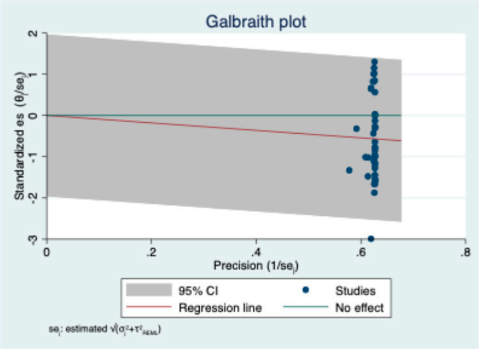

Each point represents a study.
The x-axis is the study's precision (1/SE).
The y-axis is the standardized effect size:
The central horizontal line (z = 0) represents the pooled effect.
Studies should cluster around it if there's no heterogeneity.
The funnel-shaped boundaries (typically ±2) show the 95% CI limits under homogeneity.
Points far above or below the funnel might be outliers.

Here many points outside funnel confirming massive heterogeneity. Nearly uniform precision because

- A similar sample size across studies
- Very similar weights in the random-effects model
- And even high tau^2, which dominates over SE differences

9. Sub-group analysis

```
. meta summarize, random(reml) predinterval() subgroup(locationprovince)

  Effect-size label: Effect size
        Effect size: logmean
          Std. err.: logse

Subgroup meta-analysis summary              Number of studies =     43
Random-effects model
Method: REML
Group: locationprovince

--------------------------------------------------------------------
           Study |    Effect size    [95% conf. interval]  % weight
-----------------+--------------------------------------------------
Group: Kampong C~m|
        Study 14 |         -2.501      -2.512      -2.490      2.36
        Study 15 |         -2.564      -2.621      -2.507      2.35
        Study 41 |         -0.703      -1.012      -0.395      2.33
                 |
           theta |         -1.932      -3.118      -0.745
-----------------+--------------------------------------------------
Group: Kandal    |
        Study  1 |          1.322       0.980       1.663      2.33
        Study  2 |          1.839       1.602       2.076      2.34
        Study  3 |          1.348       1.223       1.473      2.35
        Study  4 |          2.077       1.915       2.239      2.35
        Study  5 |          1.603       1.330       1.877      2.34
        Study  6 |         -1.514      -1.615      -1.413      2.35
        Study  7 |          1.639       1.410       1.868      2.34
        Study  8 |          1.314       1.056       1.572      2.34
        Study  9 |          0.896       0.868       0.924      2.36
        Study 10 |         -1.565      -1.620      -1.511      2.35
        Study 11 |         -1.911      -1.934      -1.887      2.36
        Study 43 |          1.041       0.544       1.538      2.30
                 |
           theta |          0.670      -0.153       1.493
-----------------+--------------------------------------------------
Group: Kratie    |
        Study 12 |          1.071       0.589       1.553      2.30
        Study 13 |         -2.453      -2.474      -2.433      2.36
        Study 42 |         -4.828      -5.318      -4.338      2.30
                 |
           theta |         -2.071      -5.422       1.280
-----------------+--------------------------------------------------
Group: Tonle Sap |
        Study 16 |         -2.408      -3.065      -1.751      2.26
        Study 17 |         -1.347      -1.393      -1.301      2.35
        Study 18 |         -0.462      -0.506      -0.418      2.35
        Study 19 |         -0.431      -0.487      -0.374      2.35
        Study 20 |         -1.772      -2.031      -1.513      2.34
        Study 21 |         -1.609      -1.624      -1.595      2.36
```

```
         Study 22 |         -0.211      -0.219      -0.202      2.36
         Study 23 |         -2.659      -2.716      -2.602      2.35
         Study 24 |         -1.661      -2.427      -0.895      2.22
         Study 25 |         -2.303      -3.619      -0.986      2.00
         Study 26 |         -0.494      -0.545      -0.443      2.35
         Study 27 |         -1.661      -2.303      -1.019      2.26
         Study 28 |         -0.010      -0.044       0.024      2.36
         Study 29 |         -2.659      -2.861      -2.457      2.35
         Study 30 |         -1.833      -1.879      -1.786      2.35
         Study 31 |         -2.303      -2.331      -2.274      2.36
         Study 32 |          0.020      -0.047       0.087      2.35
         Study 33 |         -1.022      -1.062      -0.981      2.35
         Study 34 |          0.049       0.036       0.062      2.36
         Study 35 |         -2.040      -2.094      -1.987      2.35
         Study 36 |         -2.996      -3.192      -2.800      2.35
         Study 37 |         -0.545      -1.640       0.551      2.10
         Study 38 |         -1.238      -1.306      -1.170      2.35
         Study 39 |         -1.238      -1.248      -1.228      2.36
         Study 40 |         -2.526      -2.612      -2.439      2.35
                  |
            theta |         -1.402      -1.779      -1.025
------------------+-------------------------------------------------
Overall           |
            theta |         -0.907      -1.387      -0.428
--------------------------------------------------------------------

Heterogeneity summary
------------------------------------------------------------------------------
         Group |      df          Q    P > Q        tau2       % I2          H2
---------------+--------------------------------------------------------------
  Kampong Cham |       2     135.06   0.000        1.092      99.92     1293.75
        Kandal |      11   27158.55    0.000        2.102      99.94     1698.52
        Kratie |       2     295.47   0.000        8.729      99.65      284.20
     Tonle Sap |      24   79530.19    0.000        0.887      99.98     4589.07
---------------+--------------------------------------------------------------
       Overall |      42     2.1e+05   0.000        2.545      99.99    11230.42
------------------------------------------------------------------------------
Test of group differences: Q_b = chi2(3) = 22.42           Prob > Q_b = 0.000

. meta summarize, random(reml) predinterval() subgroup(publishedyearago)

  Effect-size label: Effect size
        Effect size: logmean
          Std. err.: logse

Subgroup meta-analysis summary            Number of studies =     43
Random-effects model
Method: REML
Group: publishedyearago

--------------------------------------------------------------------
            Study |    Effect size    [95% conf. interval]  % weight
------------------+-------------------------------------------------
Group: 6          |
         Study 16 |         -2.408      -3.065      -1.751      2.26
         Study 17 |         -1.347      -1.393      -1.301      2.35
         Study 18 |         -0.462      -0.506      -0.418      2.35
         Study 19 |         -0.431      -0.487      -0.374      2.35
         Study 20 |         -1.772      -2.031      -1.513      2.34
         Study 21 |         -1.609      -1.624      -1.595      2.36
         Study 22 |         -0.211      -0.219      -0.202      2.36
         Study 23 |         -2.659      -2.716      -2.602      2.35
         Study 24 |         -1.661      -2.427      -0.895      2.22
         Study 25 |         -2.303      -3.619      -0.986      2.00
         Study 26 |         -0.494      -0.545      -0.443      2.35
         Study 27 |         -1.661      -2.303      -1.019      2.26
         Study 28 |         -0.010      -0.044       0.024      2.36
         Study 29 |         -2.659      -2.861      -2.457      2.35
         Study 30 |         -1.833      -1.879      -1.786      2.35
         Study 31 |         -2.303      -2.331      -2.274      2.36
         Study 32 |          0.020      -0.047       0.087      2.35
         Study 33 |         -1.022      -1.062      -0.981      2.35
         Study 34 |          0.049       0.036       0.062      2.36
         Study 35 |         -2.040      -2.094      -1.987      2.35
         Study 36 |         -2.996      -3.192      -2.800      2.35
         Study 37 |         -0.545      -1.640       0.551      2.10
         Study 38 |         -1.238      -1.306      -1.170      2.35
         Study 39 |         -1.238      -1.248      -1.228      2.36
         Study 40 |         -2.526      -2.612      -2.439      2.35
                  |
            theta |         -1.402      -1.779      -1.025
------------------+-------------------------------------------------
Group: 11         |
         Study 10 |         -1.565      -1.620      -1.511      2.35
         Study 11 |         -1.911      -1.934      -1.887      2.36
         Study 12 |          1.071       0.589       1.553      2.30
         Study 13 |         -2.453      -2.474      -2.433      2.36
         Study 14 |         -2.501      -2.512      -2.490      2.36
         Study 15 |         -2.564      -2.621      -2.507      2.35
         Study 41 |         -0.703      -1.012      -0.395      2.33
         Study 42 |         -4.828      -5.318      -4.338      2.30
         Study 43 |          1.041       0.544       1.538      2.30
                  |
            theta |         -1.606      -2.815      -0.398
```

```
-----------------+------------------------------------------------
Group: 14        |
         Study  1 |          1.322       0.980       1.663      2.33
         Study  2 |          1.839       1.602       2.076      2.34
         Study  3 |          1.348       1.223       1.473      2.35
         Study  4 |          2.077       1.915       2.239      2.35
         Study  5 |          1.603       1.330       1.877      2.34
         Study  6 |         -1.514      -1.615      -1.413      2.35
         Study  7 |          1.639       1.410       1.868      2.34
         Study  8 |          1.314       1.056       1.572      2.34
         Study  9 |          0.896       0.868       0.924      2.36
                  |
            theta |          1.166       0.467       1.865
-----------------+------------------------------------------------
Overall           |
            theta |         -0.907      -1.387      -0.428
-------------------------------------------------------------------

Heterogeneity summary
----------------------------------------------------------------------------
         Group |      df          Q    P > Q        tau2       % I2          H2
---------------+------------------------------------------------------------
             6 |      24    79530.19   0.000       0.887      99.98     4589.07
            11 |       8     3530.61   0.000       3.400      99.99    11928.21
            14 |       8     2561.18   0.000       1.132      99.63      266.89
---------------+------------------------------------------------------------
       Overall |      42     2.1e+05   0.000       2.545      99.99    11230.42
----------------------------------------------------------------------------
Test of group differences: Q_b = chi2(2) = 41.69           Prob > Q_b = 0.000

. meta summarize, random(reml) predinterval() subgroup(analysismethod)

  Effect-size label: Effect size
        Effect size: logmean
          Std. err.: logse

Subgroup meta-analysis summary            Number of studies =     43
Random-effects model
Method: REML
Group: analysismethod

-------------------------------------------------------------------
            Study |     Effect size    [95% conf. interval]  % weight
-----------------+------------------------------------------------
Group: HPLC/ICP-MS|
         Study  1 |          1.322       0.980       1.663      2.33
         Study  2 |          1.839       1.602       2.076      2.34
         Study  3 |          1.348       1.223       1.473      2.35
         Study  4 |          2.077       1.915       2.239      2.35
         Study  5 |          1.603       1.330       1.877      2.34
         Study  6 |         -1.514      -1.615      -1.413      2.35
         Study  7 |          1.639       1.410       1.868      2.34
         Study  8 |          1.314       1.056       1.572      2.34
                  |
            theta |          1.201       0.412       1.990
-----------------+------------------------------------------------
Group: ICP-MS     |
         Study  9 |          0.896       0.868       0.924      2.36
         Study 10 |         -1.565      -1.620      -1.511      2.35
         Study 11 |         -1.911      -1.934      -1.887      2.36
         Study 12 |          1.071       0.589       1.553      2.30
         Study 13 |         -2.453      -2.474      -2.433      2.36
         Study 14 |         -2.501      -2.512      -2.490      2.36
         Study 15 |         -2.564      -2.621      -2.507      2.35
         Study 16 |         -2.408      -3.065      -1.751      2.26
         Study 17 |         -1.347      -1.393      -1.301      2.35
         Study 18 |         -0.462      -0.506      -0.418      2.35
         Study 19 |         -0.431      -0.487      -0.374      2.35
         Study 20 |         -1.772      -2.031      -1.513      2.34
         Study 21 |         -1.609      -1.624      -1.595      2.36
         Study 22 |         -0.211      -0.219      -0.202      2.36
         Study 23 |         -2.659      -2.716      -2.602      2.35
         Study 24 |         -1.661      -2.427      -0.895      2.22
         Study 25 |         -2.303      -3.619      -0.986      2.00
         Study 26 |         -0.494      -0.545      -0.443      2.35
         Study 27 |         -1.661      -2.303      -1.019      2.26
         Study 28 |         -0.010      -0.044       0.024      2.36
         Study 29 |         -2.659      -2.861      -2.457      2.35
         Study 30 |         -1.833      -1.879      -1.786      2.35
         Study 31 |         -2.303      -2.331      -2.274      2.36
         Study 32 |          0.020      -0.047       0.087      2.35
         Study 33 |         -1.022      -1.062      -0.981      2.35
         Study 34 |          0.049       0.036       0.062      2.36
         Study 35 |         -2.040      -2.094      -1.987      2.35
         Study 36 |         -2.996      -3.192      -2.800      2.35
         Study 37 |         -0.545      -1.640       0.551      2.10
         Study 38 |         -1.238      -1.306      -1.170      2.35
         Study 39 |         -1.238      -1.248      -1.228      2.36
         Study 40 |         -2.526      -2.612      -2.439      2.35
                  |
            theta |         -1.383      -1.763      -1.002
-----------------+------------------------------------------------
Group: ICP-OES    |
         Study 41 |         -0.703      -1.012      -0.395      2.33
```

```
        Study 42 |         -4.828      -5.318      -4.338      2.30
        Study 43 |          1.041       0.544       1.538      2.30
                 |
           theta |         -1.496      -4.902       1.911
-----------------+---------------------------------------------------
Overall          |
           theta |         -0.907      -1.387      -0.428
---------------------------------------------------------------------

Heterogeneity summary
------------------------------------------------------------------------------
          Group |      df          Q   P > Q        tau2       % I2          H2
----------------+-------------------------------------------------------------
   HPLC/ICP-MS |       7    2414.75   0.000       1.284      99.34      152.30
        ICP-MS |      31    2.1e+05   0.000       1.173      99.99     6962.52
       ICP-OES |       2     298.53   0.000       9.011      99.48      191.70
----------------+-------------------------------------------------------------
       Overall |      42    2.1e+05   0.000       2.545      99.99    11230.42
------------------------------------------------------------------------------
Test of group differences: Q_b = chi2(2) = 33.52           Prob > Q_b = 0.000

. meta summarize, random(reml) predinterval() subgroup(samplingsite)

  Effect-size label: Effect size
        Effect size: logmean
          Std. err.: logse

Subgroup meta-analysis summary            Number of studies =     43
Random-effects model
Method: REML
Group: samplingsite

---------------------------------------------------------------------
           Study |    Effect size    [95% conf. interval]  % weight
-----------------+---------------------------------------------------
Group: Burrow pits|
        Study  3 |          1.348       1.223       1.473      2.35
        Study  4 |          2.077       1.915       2.239      2.35
        Study  5 |          1.603       1.330       1.877      2.34
        Study  8 |          1.314       1.056       1.572      2.34
                 |
           theta |          1.589       1.235       1.943
-----------------+---------------------------------------------------
Group: Controlle~a|
        Study  1 |          1.322       0.980       1.663      2.33
                 |
           theta |          1.322       0.980       1.663
-----------------+---------------------------------------------------
Group: Fish pond |
        Study  9 |          0.896       0.868       0.924      2.36
                 |
           theta |          0.896       0.868       0.924
-----------------+---------------------------------------------------
Group: Lake      |
        Study 16 |         -2.408      -3.065      -1.751      2.26
        Study 17 |         -1.347      -1.393      -1.301      2.35
        Study 18 |         -0.462      -0.506      -0.418      2.35
        Study 19 |         -0.431      -0.487      -0.374      2.35
        Study 20 |         -1.772      -2.031      -1.513      2.34
        Study 21 |         -1.609      -1.624      -1.595      2.36
        Study 22 |         -0.211      -0.219      -0.202      2.36
        Study 23 |         -2.659      -2.716      -2.602      2.35
        Study 24 |         -1.661      -2.427      -0.895      2.22
        Study 25 |         -2.303      -3.619      -0.986      2.00
        Study 26 |         -0.494      -0.545      -0.443      2.35
        Study 27 |         -1.661      -2.303      -1.019      2.26
        Study 28 |         -0.010      -0.044       0.024      2.36
        Study 29 |         -2.659      -2.861      -2.457      2.35
        Study 30 |         -1.833      -1.879      -1.786      2.35
        Study 31 |         -2.303      -2.331      -2.274      2.36
        Study 32 |          0.020      -0.047       0.087      2.35
        Study 33 |         -1.022      -1.062      -0.981      2.35
        Study 34 |          0.049       0.036       0.062      2.36
        Study 35 |         -2.040      -2.094      -1.987      2.35
        Study 36 |         -2.996      -3.192      -2.800      2.35
        Study 37 |         -0.545      -1.640       0.551      2.10
        Study 38 |         -1.238      -1.306      -1.170      2.35
        Study 39 |         -1.238      -1.248      -1.228      2.36
        Study 40 |         -2.526      -2.612      -2.439      2.35
                 |
           theta |         -1.402      -1.779      -1.025
-----------------+---------------------------------------------------
Group: Large fis~m|
        Study  6 |         -1.514      -1.615      -1.413      2.35
                 |
           theta |         -1.514      -1.615      -1.413
-----------------+---------------------------------------------------
Group: Natural h~t|
        Study 10 |         -1.565      -1.620      -1.511      2.35
        Study 11 |         -1.911      -1.934      -1.887      2.36
        Study 12 |          1.071       0.589       1.553      2.30
        Study 13 |         -2.453      -2.474      -2.433      2.36
        Study 14 |         -2.501      -2.512      -2.490      2.36
        Study 15 |         -2.564      -2.621      -2.507      2.35
```

```
                  |
            theta |         -1.668       -2.761       -0.576
------------------+--------------------------------------------------
Group: Natural w~d|
         Study  2 |          1.839        1.602        2.076      2.34
                  |
            theta |          1.839        1.602        2.076
------------------+--------------------------------------------------
Group: Pond &river|
         Study 41 |         -0.703       -1.012       -0.395      2.33
         Study 42 |         -4.828       -5.318       -4.338      2.30
         Study 43 |          1.041        0.544        1.538      2.30
                  |
            theta |         -1.496       -4.902        1.911
------------------+--------------------------------------------------
Group: Small fis~m|
         Study  7 |          1.639        1.410        1.868      2.34
                  |
            theta |          1.639        1.410        1.868
------------------+--------------------------------------------------
Overall           |
            theta |         -0.907       -1.387       -0.428
---------------------------------------------------------------------

Heterogeneity summary
------------------------------------------------------------------------------
         Group |       df          Q   P > Q        tau2       % I2        H2
---------------+--------------------------------------------------------------
   Burrow pits |        3      53.35   0.000       0.119      92.82      13.93
Controlled a~a |        0       0.00       .       0.000          .          .
     Fish pond |        0       0.00       .       0.000          .          .
          Lake |       24   79530.19   0.000       0.887      99.98    4589.07
Large fish f~m |        0      -0.00       .       0.000          .          .
Natural habi~t |        5    3137.45   0.000       1.853      99.99   10365.55
Natural wetl~d |        0       0.00       .       0.000          .          .
   Pond &river |        2     298.53   0.000       9.011      99.48     191.70
Small fish f~m |        0       0.00       .       0.000          .          .
---------------+--------------------------------------------------------------
       Overall |       42    2.1e+05   0.000       2.545      99.99   11230.42
------------------------------------------------------------------------------
Test of group differences: Q_b = chi2(8) = 2358.05         Prob > Q_b = 0.000
```

10. Meta-regression

Very high heterogeneity warrants sub-group analysis and meta-regression to understand

Year published – only three variables so not at all normal. Try dichotomising and also log transformation

First individual predictors

Predictor	**Coefficient (β)**	**p-value**	**R^2 (%)**	**Interpretation (Back-transformed if relevant)**	Include in model
Paper id (**paperno**)	–0.33	<0.001	20.9%	Later-numbered papers report lower arsenic. A random effect or grouping variable	No RE
Sample size (**samplesize**)	–0.17	0.001	0.0%	Larger studies report lower arsenic. Explains 0% - probably a confounder	No confounder
Years ago published (**publishedyearago**)	–0.059	0.040	0.0%	More recent studies report higher arsenic	Small effect 0 r2
Fish sampled (**fish**)	+1.38	0.009	19.3%	Arsenic ~**4.0× higher** in fish vs. other aquatic animals (exp(1.38))	Yes
Artificial site (**artificial**)	+2.45	<0.001	35.4%	Arsenic ~**11.6× higher** in artificial sites (exp(2.45))	Yes
Method: ICP-MS (**icp_ms**)	–1.86	<0.001	25.0%	Arsenic ~**6.4× lower** than reference (exp(–1.86))	No redundant
Method: HPLC/ICP-MS (**hplcicpms**)	+2.59	<0.001	40.0%	Arsenic ~**13.3× higher** (exp(2.59))	yes
Method: ICP-OES (**icp_oes**)	–0.63	0.517	0.0%	Not significant	No redundant
Province: Tonle Sap (**tonlesap**)	–1.19	0.011	11.7%	Arsenic ~**70% lower** (exp(–1.19) ≈ 0.30×)	yes
Province: Kandal (**kandal**)	+2.19	<0.001	37.4%	Arsenic ~**8.9× higher** (exp(2.19))	yes
Province: Kratie (**kratie**)	–1.25	0.189	1.8%	Not significant	No

Province: Kampong Cham (**kampong**)	–1.10	0.250	0.8%	Not significant	

Goal of Multivariable Meta-Regression
To jointly model key study-level predictors that explain heterogeneity in arsenic concentrations, while:
Adjusting for potential confounding

Avoiding overfitting (especially with 43 studies)
Selecting interpretable and non-redundant variables

the biggest category as reference (don't include)

- It's the most statistically **stable** baseline
- It avoids comparison across tiny groups (e.g., comparing two provinces that both have 3 observations is unreliable)
- allows you to meaningfully interpret differences *relative to the most common setting* — which reviewers also prefer

```
. meta regress artificial hplcicpms fish tonlesap kandal, random(reml))


Effect-size label: Effect size
Effect size: logmean
Std. err.: logse

Random-effects meta-regression          Number of obs  =        43
Method: REML        Residual heterogeneity:
             tau2 =    1.43
             I2 (%) =   99.98
             H2 = 5547.57
             R-squared (%) =   43.83
             Wald chi2(5)    =    36.99
             Prob > chi2     =    0.0000

_meta_es  Coefficient  Std. err.        z      P>z     [95% conf. interval]

artificial    .2398544   .9482065       0.25   0.800    -1.618596    2.098305
hplcicpms    1.179771   .9470724       1.25   0.213    -.6764573    3.035998
fish         -1.100854   .7291629       -1.51  0.131    -2.529987    .3282786
tonlesap    .5948976   .5487634        1.08   0.278    -.4806588    1.670454
kandal       1.262254   .8273345        1.53   0.127    -.3592921      2.8838
_cons        -.9003257   .8797755       -1.02  0.306    -2.624654    .8240027

Test of residual homogeneity: Q_res =   chi2(37)      = 81460.75Prob > Q_res =
0.0000
```

11. Publication bias and small study effect

```
. meta trimfill

  Effect-size label: Effect size
        Effect size: logmean
          Std. err.: logse

Nonparametric trim-and-fill analysis of publication bias
Linear estimator, imputing on the right

Iteration                          Number of studies =     46
  Model: Random-effects                     observed =     46
 Method: REML                               imputed =      0
```

```
Pooling
  Model: Random-effects
 Method: REML

---------------------------------------------------------------
             Studies |      Effect size    [95% conf. interval]
---------------------+-----------------------------------------
            Observed |           -1.119      -1.123      -1.114
  Observed + Imputed |           -1.119      -1.123      -1.114
---------------------------------------------------------------

. meta bias, egger random(reml)

  Effect-size label: Effect size
        Effect size: logmean
          Std. err.: logse

Regression-based Egger test for small-study effects
Random-effects model
Method: REML

H0: beta1 = 0; no small-study effects
            beta1 =     -0.36
      SE of beta1 =     1.683
                z =     -0.22
       Prob > |z| =    0.8297
```

References

Hozo, S. P., Djulbegovic, B., & Hozo, I. (2005). Estimating the mean and variance from the median, range, and the size of a sample. *BMC Medical Research Methodology*, *5*. https://doi.org/10.1186/1471-2288-5-13

Walter, S. D., & Yao, X. (2007). Effect sizes can be calculated for studies reporting ranges for outcome variables in systematic reviews. *Journal of Clinical Epidemiology*, *60*(8), 849–852. https://doi.org/10.1016/j.jclinepi.2006.11.003

Weir, C. J., Butcher, I., Assi, V., Lewis, S. C., Murray, G. D., Langhorne, P., & Brady, M. C. (2018). Dealing with missing standard deviation and mean values in meta-analysis of continuous outcomes: A systematic review. In *BMC Medical Research Methodology* (Vol. 18, Issue 1). BioMed Central Ltd. https://doi.org/10.1186/s12874-018-0483-0
