# Supplementary material for: Chemical Hazards in Products of Animal Origin in Cambodia from 2000 to 2023: A Systematic Review and Meta-Analysis
Source: Int J Environ Res Public Health. 2025 Aug 19;22(8):1299. doi: 10.3390/ijerph22081299 (PMC12386664; doi:10.3390/ijerph22081299)
Supplement: Supplementary file 1 [file ijerph-22-01299-s001.zip › Supplementary material S2.pdf]

Appendix A: Supplementary material 1-List of published papers and reports included in the review

Ref. No.	Full reference
Paper 1	Murphy, T., Sampson, M., Le, X., Irvine, K., Gerads, R., Smith, L. and Parr, T. (2010) Arsenic Bioaccumulation in an Arsenic Rich area of Cambodia, In Irvine, K., Murphy, T., Vermette, S., and Vanchan, V. (eds.), *Water Resources and Development in Southeast Asia*, Boston, MA, Pearson Custom Publishing, pp. 57–88.
Paper 2	Kongkea Phan. and Kyoung-Woong Kim (2018) Arsenic health risk in foodstuffs in the Mekong River basin of Cambodia, In *5th International Arsenic Symposium*, Miyazaki, Japan, Faculty of Science and technology, International University, Phnom Penh.
Paper 3	Ramu, K., Kajiwara, N., Sudaryanto, A., Isobe, T., Takahashi, S., Subramanian, A., Ueno, D., Zheng, G. J., Lam, P. K. S., Takada, H., Zakaria, M. P., Viet, P. H., Prudente, M., Tana, T. S. and Tanabe, S. (2007) Asian Mussel Watch Program: Contamination Status of Polybrominated Diphenyl Ethers and Organochlorines in Coastal Waters of Asian Countries, *Environmental Science & Technology*, 41(13), pp. 4580–4586.
Paper 4	Sudaryanto, A., Takahashi, S., Monirith, I., Ismail, A., Muchtar, M., Zheng, J., Richardson, B. J., Subramanian, A., Prudente, M., Hue, N. D. and Tanabe, S. (2002) Asia-Pacific mussel watch: Monitoring of butyltin contamination in coastal waters of Asian developing countries, *Environmental Toxicology and Chemistry*, 21(10), pp. 2119–2130.
Paper 5	Douny, C., Mith, H., Igout, A. and Scippo, M.-L. (2021) Fatty acid intake, biogenic amines and polycyclic aromatic hydrocarbons exposure through the consumption of nine species of smoked freshwater fish from Cambodia, *Food Control*, 130, p. 108219.
Paper 6	Cheng, Z., Li, H.-H., Wang, H., Zhu, X.-M., Sthiannopkao, S., Kim, K.-W., Yasin, M. S. M., Hashim, J. H. and Wong, M.-H. (2016) Dietary exposure and human risk assessment of phthalate esters based on total diet study in Cambodia, *Environmental Research*, 150, pp. 423–430.
Paper 7	Cheng, Z., Wang, H.-S., Du, J., Sthiannopkao, S., Xing, G.-H., Kim, K.-W., Yasin, M. S. M., Hashim, J. H. and Wong, M.-H. (2013) Dietary exposure and risk assessment of mercury via total diet study in Cambodia, *Chemosphere*, 92(1), pp. 143–149.
Paper 8	Isobe, T., Takada, H., Kanai, M., Tsutsumi, S., Isobe, K. O., Boonyatumanond, R. and Zakaria, M. P. (2007) Distribution of Polycyclic Aromatic Hydrocarbons (PAHs) and phenolic endocrine disrupting chemicals in South and Southeast Asian mussels, *Environmental Monitoring and Assessment*, 135(1–3), pp. 423–440.
Paper 9	Agusa, T., Kunito, T., Sudaryanto, A., Monirith, I., Kan-Atireklap, S., Iwata, H., Ismail, A., Sanguansin, J., Muchtar, M., Tana, T. S. and Tanabe, S. (2007) Exposure assessment for trace elements from consumption of marine fish in Southeast Asia, *Environmental Pollution*, 145(3), pp. 766–777.
Paper 10	Kelly, B. C., Myo, A. N., Pi, N., Bayen, S., Leakhena, P. C., Chou, M. and Tan, B. H. (2018) Human exposure to trace elements in central Cambodia: Influence of seasonal hydrology and food-chain bioaccumulation behaviour, *Ecotoxicology and Environmental Safety*, 162, pp. 112–120.
Paper 11	Slámová, T., Fraňková, A. and Banout, J. (2021) Influence of traditional Cambodian smoking practices on the concentration of Polycyclic Aromatic Hydrocarbons (PAHs) in smoked fish processed in the Tonle Sap area, Cambodia, *Journal of Food Composition and Analysis*, 100, p. 103902.
Paper 12	Murphy, T. and Irvine, K. (2009) Mercury contamination along the Mekong River, Cambodia, *Environmental Science*, 6, pp. 1–9.

Paper 13	Slámová, T., Fraňková, A., Hubáčková, A. and Banout, J. (2017) Polycyclic aromatic hydrocarbons in Cambodian smoked fish, *Food Additives & Contaminants: Part B*, pp. 1–8.
Paper 14	Basri, D. F., Abu Bakar, N. F., Fudholi, A., Ruslan, M. H. and Saroeun, I. (2015) Comparison of Selected Metals Content in Cambodian Striped Snakehead Fish (*Channa striata*) Using Solar Drying System and Open Sun Drying, *Journal of Environmental and Public Health*, 2015, pp. 1–6.
Paper 15	Wang, H.-S., Sthiannopkao, S., Chen, Z.-J., Man, Y.-B., Du, J., Xing, G.-H., Kim, K.-W., Mohamed Yasin, M. S., Hashim, J. H. and Wong, M.-H. (2013) Arsenic concentration in rice, fish, meat and vegetables in Cambodia: a preliminary risk assessment, *Environmental Geochemistry and Health*, 35(6), pp. 745–755.
Paper 16	Phan, K., Sthiannopkao, S., Heng, S., Phan, S., Huoy, L., Wong, M. H. and Kim, K.-W. (2013) Arsenic contamination in the food chain and its risk assessment of populations residing in the Mekong River basin of Cambodia, *Journal of Hazardous Materials*, 262, pp. 1064–1071.
Paper 17	Wang, H.-S., Sthiannopkao, S., Du, J., Chen, Z.-J., Kim, K.-W., Mohamed Yasin, M. S., Hashim, J. H., Wong, C. K.-C. and Wong, M.-H. (2011) Daily intake and human risk assessment of organochlorine pesticides (OCPs) based on Cambodian market basket data, *Journal of Hazardous Materials*, 192(3), pp. 1441–1449.
Paper 18	Marcussen, H., Dalsgaard, A. and Holm, P. E. (2009) Element concentrations in water spinach (*Ipomoea aquatica* Forssk.), fish and sediment from a wetland production system that receives wastewater from Phnom Penh, Cambodia, *Journal of Environmental Science and Health, Part A*, 44(1), pp. 67–77.
Paper 19	Agusa, T., Kunito, T., Iwata, H., Monirith, I., Tana, T. S., Subramanian, A. and Tanabe, S. (2005) Mercury contamination in human hair and fish from Cambodia: levels, specific accumulation and risk assessment, *Environmental Pollution*, 134(1), pp. 79–86.
Report 1	National Residue Monitoring Plan report by Fisheries Administration, Phnom Penh, Cambodia (2020)
Report 2	National Residue Monitoring Plan report by Fisheries Administration, Phnom Penh, Cambodia (2021)
Report 3	National Residue Monitoring Plan report by Fisheries Administration, Phnom Penh, Cambodia (2022)
Report 4	National Residue Monitoring Plan report by Fisheries Administration, Phnom Penh, Cambodia (2023)
