# Supplementary material for: Chemical Hazards in Products of Animal Origin in Cambodia from 2000 to 2023: A Systematic Review and Meta-Analysis
Source: Int J Environ Res Public Health. 2025 Aug 19;22(8):1299. doi: 10.3390/ijerph22081299 (PMC12386664; doi:10.3390/ijerph22081299)

Mercury Fish

A pooled meta-analysis can still be done with 4 papers, if:

- The studies are reasonably homogeneous, or heterogeneity is estimable (I^2, τ^2)
- You're using a random-effects model to reflect between-study variability

However:

- Estimates of between-study variance (τ^2) will be unstable with few studies.
- Confidence intervals will be wide, and prediction intervals may be very broad.

So a pooled estimate is feasible, but interpret it with caution.

Meta-regression needs at least 10 studies per predictor to avoid overfitting and spurious associations.
With 4 papers, you don't have enough degrees of freedom to reliably estimate covariate effects.

```
. gen se= sd/sqrt(Samplenumber_n)
. hist mean_mercury
. qnorm mean_mercury

ladder mean_mercury

Transformation         Formula              chi2(2)  Prob > chi2
-----------------------------------------------------------------
Cubic                  mean_m~y^3             28.80        0.000
Square                 mean_m~y^2             16.68        0.000
Identity               mean_m~y                5.29        0.071
Square root            sqrt(mean_m~y)          4.77        0.092
Log                    log(mean_m~y)           1.73        0.420
1/(Square root)        1/sqrt(mean_m~y)       12.14        0.002
Inverse                1/mean_m~y             23.53        0.000
1/Square               1/(mean_m~y^2)         32.64        0.000
1/Cubic                1/(mean_m~y^3)         36.14        0.000

. generate logmean = log(mean_mercury)
. generate logse = se /mean_mercury

. hist logmean
(bin=5, start=-4.6051702, width=.90435773)
. qnorm logmean

. meta set logmean logse

Meta-analysis setting information

 Study information
    No. of studies: 31
       Study label: Generic
        Study size: N/A

       Effect size
              Type: <generic>
             Label: Effect size
          Variable: logmean

         Precision
         Std. err.: logse
                CI: [_meta_cil, _meta_ciu]
          CI level: 95%

  Model and method
             Model: Random effects
            Method: REML
```

```
. meta summarize, random(reml) predinterval(95) se(khartung)

  Effect-size label: Effect size
        Effect size: logmean
          Std. err.: logse

Meta-analysis summary                     Number of studies =     31
Random-effects model                      Heterogeneity:
Method: REML                                          tau2 =  1.4070
SE adjustment: Knapp-Hartung                        I2 (%) =   95.14
                                                        H2 =   20.56

--------------------------------------------------------------------
           Study |    Effect size    [95% conf. interval]  % weight
-----------------+--------------------------------------------------
        Study  1 |         -2.659      -3.356      -1.963      3.59
        Study  2 |         -1.897      -4.591       0.797      1.67
        Study  3 |         -2.813      -5.998       0.372      1.36
        Study  4 |         -0.478      -1.442       0.486      3.34
        Study  5 |         -2.659      -3.917      -1.402      3.03
        Study  6 |         -1.561      -2.584      -0.537      3.28
        Study  7 |         -0.616      -1.779       0.547      3.13
        Study  8 |         -0.431      -1.260       0.398      3.47
        Study  9 |         -0.494      -1.380       0.391      3.41
        Study 10 |         -1.833      -2.492      -1.173      3.62
        Study 11 |         -2.659      -3.534      -1.784      3.43
        Study 12 |         -1.514      -2.680      -0.349      3.13
        Study 13 |         -1.897      -2.937      -0.858      3.26
        Study 14 |         -2.659      -3.699      -1.620      3.26
        Study 15 |         -0.942      -1.859      -0.024      3.38
        Study 16 |         -2.526      -3.950      -1.101      2.84
        Study 17 |         -1.079      -1.961      -0.197      3.42
        Study 18 |         -0.357      -1.495       0.782      3.15
        Study 19 |         -0.528      -1.482       0.427      3.35
        Study 20 |         -0.083      -1.213       1.046      3.16
        Study 21 |         -3.689      -4.244      -3.134      3.70
        Study 22 |         -1.833      -2.374      -1.292      3.71
        Study 23 |         -4.457      -4.975      -3.939      3.73
        Study 24 |         -2.303      -2.439      -2.166      3.90
        Study 25 |         -0.693      -0.868      -0.518      3.89
        Study 26 |         -2.659      -3.035      -2.284      3.81
        Study 27 |         -4.605      -5.482      -3.729      3.42
        Study 28 |         -3.219      -3.502      -2.936      3.85
        Study 29 |         -3.912      -5.609      -2.215      2.55
        Study 30 |         -1.204      -2.939       0.531      2.51
        Study 31 |         -2.996      -4.580      -1.412      2.67
-----------------+--------------------------------------------------
           theta |         -1.969      -2.437      -1.502
--------------------------------------------------------------------
95% prediction interval for theta: [-4.440, 0.501]

Test of theta = 0: t(30) = -8.60                Prob > |t| = 0.0000
Test of homogeneity: Q = chi2(30) = 567.00        Prob > Q = 0.0000

. display exp(-1.969)
.13959638
```

The average mercury level in fish is ~0.14 µg/g (95% CI: 0.087 to 0.223), significantly above zero and likely meaningful for risk or regulation.

Large τ^2 suggesting large between-study variance (log scale) and high I^2 95% of total variability is between studies. Cochran's Q confirms significant heterogeneity.

There is substantial unexplained variability across studies, suggesting differences in species, sites, methods, or other study-level characteristics.

In a future study, the mercury level could plausibly fall anywhere between **0.012 and 1.65 µg/g** — a very wide range, consistent with the high heterogeneity.

□ Most studies carry **~3% weight**, indicating a balanced sample.
□ No single study dominates the result, but the high τ^2 reduces precision.

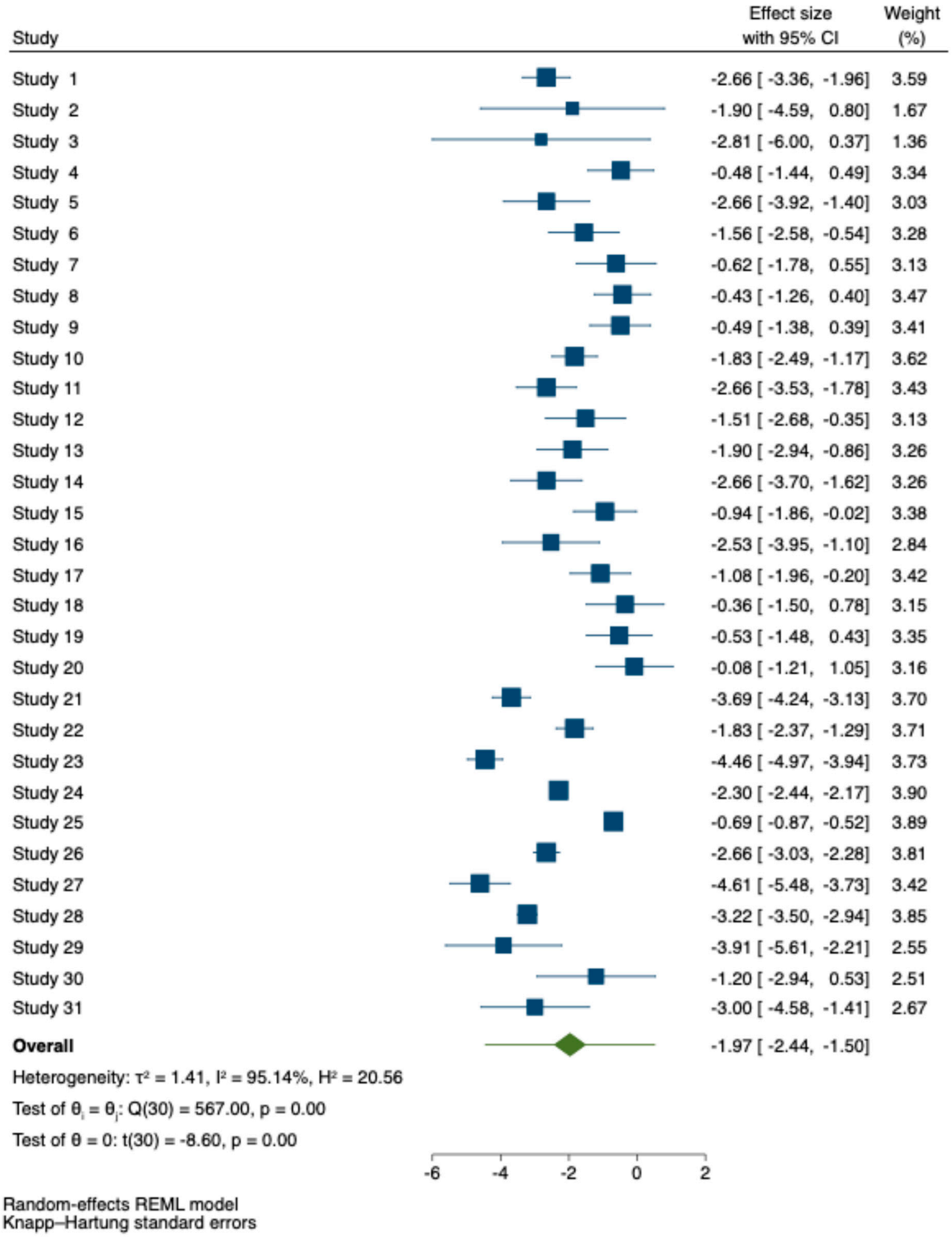

Supplement: Supplementary file 1 [file ijerph-22-01299-s001.zip › Supplementary material S3.pdf]
